# Prenatal inflammation impairs early CD11c-positive microglia induction and delays myelination in neurodevelopmental disorders

**DOI:** 10.1038/s42003-025-07511-3

**Published:** 2025-01-17

**Authors:** Kazuya Fuma, Yukako Iitani, Kenji Imai, Takafumi Ushida, Sho Tano, Kosuke Yoshida, Akira Yokoi, Rika Miki, Hiroyuki Kidokoro, Yoshiaki Sato, Yuichiro Hara, Tomoo Ogi, Kohei Nomaki, Makoto Tsuda, Okiru Komine, Koji Yamanaka, Hiroaki Kajiyama, Tomomi Kotani

**Affiliations:** 1 Department of Obstetrics and Gynecology, Nagoya University Graduate School of Medicine, Nagoya, Japan; 2 Division of Reproduction and Perinatology, Center for Maternal-Neonatal Care, Nagoya University Hospital, Nagoya, Japan; 3 Laboratory of Bell Research Center‑Department of Obstetrics and Gynecology Collaborative Research, Nagoya University Graduate School of Medicine, Nagoya, Japan; 4 Department of Pediatrics, Nagoya University Graduate School of Medicine, Nagoya, Japan; 5 Division of Neonatology, Center for Maternal-Neonatal Care, Nagoya University Hospital, Nagoya, Japan; 6 Department of Genetics, Research Institute of Environmental Medicine, Nagoya University, Nagoya, Japan; 7 Department of Human Genetics and Molecular Biology, Nagoya University Graduate School of Medicine, Nagoya, Japan; 8 Institute for Glyco-core Research (iGCORE), Nagoya University, Nagoya, Japan; 9 Center for One Medicine Innovative Translational Research (COMIT), Nagoya University, Nagoya, Japan; 10 Department of Molecular and System Pharmacology, Graduate School of Pharmaceutical Sciences, Kyushu University, Fukuoka, Japan; 11 Kyushu University Institute for Advanced Study, Fukuoka, Japan; 12 Department of Neuroscience and Pathobiology, Nagoya University Graduate School of Medicine, Nagoya, Japan; 13 Department of Neuroscience and Pathobiology, Research Institute of Environmental Medicine, Nagoya University, Nagoya, Japan

**Keywords:** Molecular medicine, Microglia, Developmental biology

## Abstract

Histological chorioamnionitis (HCA) is a form of maternal immune activation (MIA) linked to an increased risk of neurodevelopmental disorders in offspring. Our previous study identified neurodevelopmental impairments in an MIA mouse model mimicking HCA. Thus, this study investigated the role of CD11c^+^ microglia, key contributors to myelination through IGF-1 production, in this pathology. In the mouse model, the CD11c^+^ microglial population was significantly lower in the MIA group than in the control group on postnatal day 3 (PN3d). Furthermore, myelination-related protein levels significantly decreased in the MIA group at PN8d. In humans, preterm infants with HCA exhibited higher IL-6 and IL-17A cord-serum levels and lower IGF-1 levels than those without HCA, followed by a higher incidence of delayed myelination on magnetic resonance imaging at the term-equivalent age. In silico analysis revealed that the transient induction of CD11c^+^ microglia during early development occurred similarly in mice and humans. Notably, a lack of high CD11c^+^ microglial population has been observed in children with neurodevelopmental disorders. This study reports impaired induction of CD11c^+^ microglia during postnatal development in a mouse model of MIA associated with delayed myelination. Our findings may inform strategies for improving outcomes in infants with HCA.

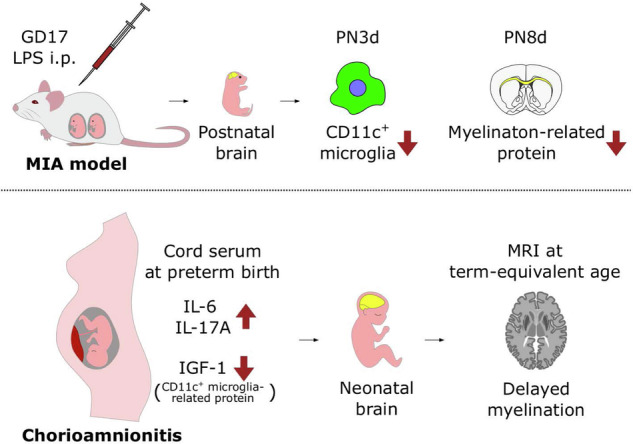

## Abstract (web summary)

CD11c-positive microglia, which have an important role in myelination through IGF-1 production during early development, are poorly induced in infants with histological chorioamnionitis and this may be involved in the development of neurodevelopmental impairments.

## Introduction

Emerging evidence supports the maternal immune activation (MIA) hypothesis, which advocates that exposure to inflammation in utero adversely affects neurodevelopment. The original theory of MIA focused only on children born to mothers who had viral infections during pregnancy. In addition, most experimental studies were conducted using poly(I:C), a Toll-like receptor-3 agonist^1,2^. However, elevated levels of interleukin-17 (IL-17), produced by T-helper 17 (Th17) cells, have been identified as the main causative factor of MIA^3,4^. Consequently, the triggers of this condition are no longer considered limited to viral infections, and the scope of MIA has been expanded to encompass several autoimmune and inflammatory disorders^5–7^.

Chorioamnionitis, the leading cause of intrauterine inflammation and preterm birth, is related to IL-17A pathology, including cerebral palsy^8^. Notably, prematurity is insufficient to cause fetal neurological aberrations, and intrauterine inflammation is necessary for such impairments^9^. A meta-analysis revealed that only histological chorioamnionitis (HCA), not clinical chorioamnionitis, poses a risk for cerebral palsy in preterm and term infants^10^. Furthermore, HCA presents long-term adverse neurodevelopmental outcomes^11–13^ and has been associated with autism spectrum disorder (ASD)^12,14^. The gram-negative endotoxin lipopolysaccharide (LPS)-induced inflammation model is commonly used as a chorioamnionitis-induced perinatal brain injury model^15–20^. Additionally, our previous findings revealed that the offspring of the LPS model exhibited pathological changes in the fetal brain^21^ and ASD-like behavioral deficits^22^. However, the underlying pathological mechanisms remain unclear.

Activated microglia are critical players in neuroinflammatory diseases. Moreover, microglia play a physiological role in neurodevelopment, including axonal growth regulation^23,24^. CD11c^+^ microglia, a major source of insulin-like growth factor (IGF)-1^25^, are critical for myelination and have been reported to significantly expand during postnatal days 3–5 (PN3*–*5 d)^26^. Proliferative region-associated microglia (PAM) express *Cd11c* and appear transiently during the first postnatal week^27^. Moreover, CD11c^+^ spiral microglia produce IGF-1 and are involved in the recovery of neuropathic pain^28^. CD11c^+^ microglia are required for developmental myelination, and the loss of CD11c^+^ microglia is associated with demyelination in multiple sclerosis^29–32^.

Currently, the effects of MIA on CD11c^+^ microglia remain unexplored. In our previous study, we identified impaired sociability and cognition, and reduced oligodendrocyte numbers in an LPS-induced MIA mouse model^21,22^. In this study, we expanded on our previous research to explore the mechanisms of poor neurodevelopment in children exposed to HCA, with a particular focus on the involvement of CD11c microglia. Here, we demonstrated that the induction of CD11c in the corpus callosum during early development is suppressed in the MIA mouse model, which may result in subsequent impaired myelination. Furthermore, we revealed the increased incidence of delayed myelination in human preterm infants exposed to HCA using magnetic resonance imaging (MRI) at a term-equivalent age.

## Results

### Decreased CD11c^+^ microglia in PN3d pups in the MIA group

**Fig. 1:**
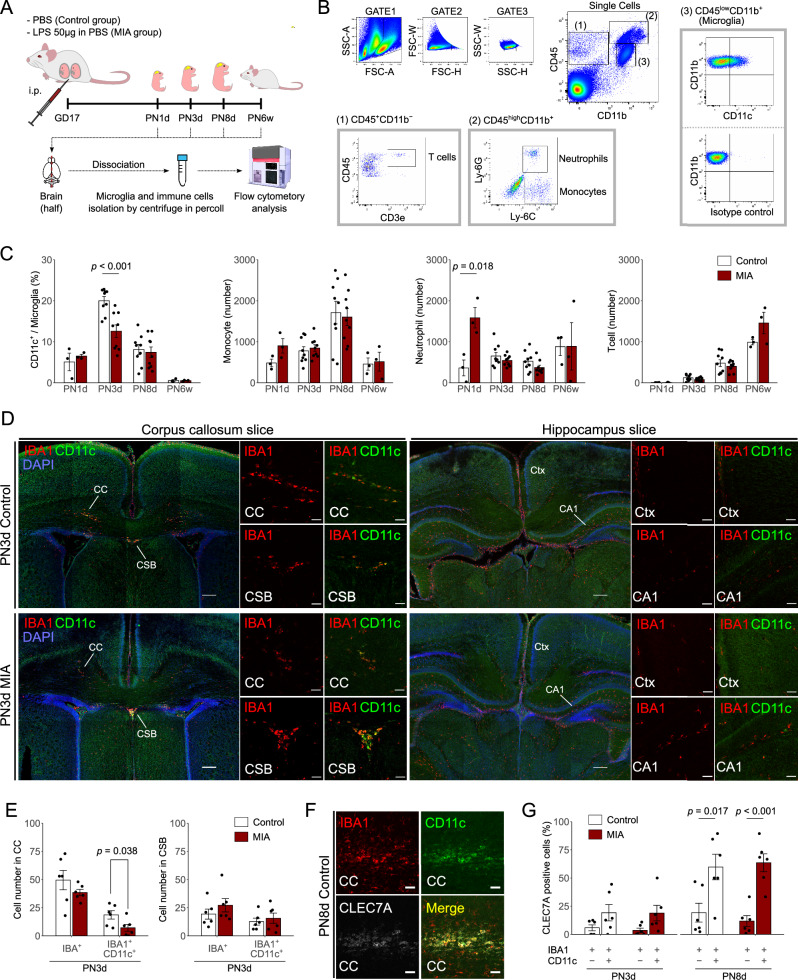
Comparison of microglial features between the control and MIA groups. **A** Protocol of the MIA modeling and cell isolation for flow cytometry analysis. The image of a flow cytometry equipment is from TogoTV (© 2016 DBCLS TogoTV, https://togotv.dbcls.jp), and the images of a conical tube and the mouse brain are from Lab icons (© K Mine, https://lab-icons.com) according to express permission from creators respectively. **B** Representative gating protocol used to sort T cells (left lower panel), neutrophils and monocytes (middle lower panel), and CD11c^+^ or CD11c^−^ microglia (right panel). Images are generated from one individual brain suspension prepared from the PN3d control group. **C** The percentage of CD11c^+^ microglia in the total microglia population, and the number of monocytes, neutrophils, and T cells in the control and MIA groups at each postnatal period (*n* = 3 in each group at PN1d and PN6w; *n* = 9 at PN3d and PN8d. Maximum 2 offspring per litter). **D** Immunofluorescence staining of IBA1 and CD11c in the corpus callosum (left panel) and hippocampus slices (right panel) in PN3d control (upper panel) and MIA offspring (lower panel). Scale bar = 200 µm (tiling image), 50 µm (magnified image). **E** Cell numbers of IBA1^+^CD11c^+^ microglia in the corpus callosum and cortico-septal boundary in PN3d offspring (control group, *n* = 6 from two litters; MIA group, *n* = 6 from three litters). **F** Immunofluorescence staining of IBA1, CD11c, and CLEC7A in the corpus callosum in the control group in PN8d offspring. Scale bar = 50 µm. **G** The percentage of CLEC7A^+^ cells among IBA1^+^CD11c^+^ or IBA1^+^ CD11c^-^ microglia in the corpus callosum in PN3d and PN8d offspring (*n* = 6 from 2 to 3 litters in each group). Data are presented as means ± SEMs. MIA maternal immune activation, CC corpus callosum, CSB cortico-septal boundary, CTX cerebral cortex, CA1 hippocampus CA1 region, SEM standard error of measurement.

The brain and body weights of the MIA group were significantly lower than those of the control group at PN3d (Supplementary Fig. 1A). We performed flow cytometric analyses of microglia from the MIA offspring to examine whether the CD11c^+^ microglial population was altered by MIA (Fig. 1A, B). The percentage of CD11c^+^ microglia among the total microglia in the MIA group was significantly lower than that in the control group at PN3d (Fig. 1C, *p* < 0.001). However, the percentage of CD11c^+^ microglia did not significantly differ between the control and MIA groups at PN1d, PN8d, and PN6w (Fig. 1C). The numbers of monocytes showed no differences at any time point in both groups (Fig. 1C). However, the number of neutrophils in the MIA group showed an approximately three-fold increase compared to the control group at PN1d (Fig. 1C, *p* = 0.018), and no difference was detected in both groups after PN3d (Fig. 1C). The number of T cells showed increasing trends as the days progressed in both the control and MIA groups (Fig. 1C).

Fluorescent immunostaining showed that CD11c^+^ microglia were present in clusters on the left and right lateral sides of the corpus callosum and in the cortico-septal boundary and were barely detectable in the cortex and hippocampus at PN3d (Fig. 1D). The number of IBA1^+^ microglia in the corpus callosum were similar between the control and MIA groups at PN3d (Fig. 1E). However, IBA1^+^CD11c^+^ cells significantly decreased in the MIA group at PN3d (Fig. 1E, *p* = 0.038), whereas those in the cortico-septal boundary did not significantly differ between the control and MIA groups. The number of IBA1^+^CD11c^+^ microglia in MIA offspring did not significantly differ between the control and MIA groups at PN8d (Supplementary Fig. 1B, C). Moreover, these IBA1^+^CD11c^+^ microglia expressed CLEC7A (a PAM marker) at a significantly higher percentage at PN8d in both the control and MIA groups (Fig. 1F, G; *p* = 0.017 and *p* < 0.001, respectively) and at a higher trend at PN3d compared to the IBA1^+^CD11c^−^ microglia (Fig. 1G).

We performed a similar analysis using *CD11c-Venus* transgenic mice to complement the staining intensity of CD11c (methods are detailed in the Supplementary Methods). Similarly, CD11c^+^ microglia were significantly fewer in the corpus callosum of PN3d offspring in the MIA group than those in the control group (*p* = 0.002, Supplementary Fig. 2). Additionally, these CD11c^+^ microglia were positive for P2Y12R, indicating that they were not infiltrating macrophages (Supplementary Fig. 2).

**Fig. 2:**
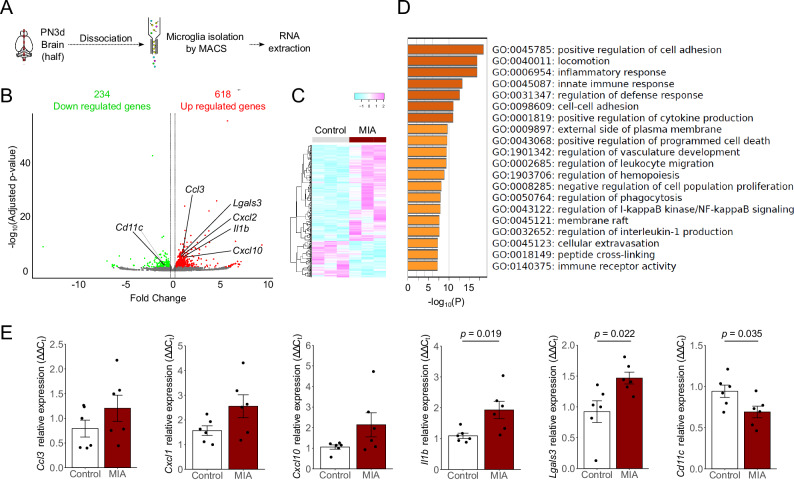
Microglial transcriptome analysis at PN3d in the control and MIA groups. **A** Protocol of microglia isolation and RNA extraction. **B** Volcano plots of DEGs in microglia isolated from the MIA group compared to the control group at PN3d (*n* = 3 offspring from 2 to 3 litters in each group). **C** Heatmap showing the gene expression levels in the microglia isolated from the control and MIA groups at PN3d. **D** GO enrichment analysis of microglia isolated from the MIA group compared to that of the control group. **E** qRT-PCR showing the relative expression of several known DEGs associated with microglial function (*n* = 6 in each group; maximum 2 offspring per litter). Data are presented as means ± SEMs. DEGs differentially expressed genes, MIA maternal immune activation, PN postnatal, SEM standard error of measurement, qRT-PCR quantitative reverse transcription-polymerase chain reaction, GO gene ontology.

RNA-seq analysis was performed to explore the distinct characteristics of microglia at PN3d in both the control and MIA groups (Fig. 2A). In total, 852 genes were identified as DEGs, of which 618 were upregulated and 234 downregulated in PN3d-microglia isolated from the MIA group (Fig. 2B). Several identified genes (*Ccl3*, *Cxcl1*, *Cxcl10*, *Il1b*, and *Lgals3*) are known to be upregulated in microglia exposed to LPS and models subjected to stress^33–37^. Heatmap analysis revealed that PN3d-microglia isolated from the MIA group showed different RNA expression profiles compared to those of the control group (Fig. 2C). Additionally, GO enrichment analysis revealed that the inflammatory response, innate immune response, leukocyte migration regulation, and phagocytosis regulation were included in the top 20 significantly altered pathways (Fig. 2D). Moreover, qRT-PCR performed to validate the results demonstrated significantly increased expression levels of *Il1b* and *Lgals3* (*p* = 0.019 and 0.022, respectively), along with a tendency towards an increase in *Ccl3, Cxcl1*, and *Cxcl10* levels in the MIA group (Fig. 2E). Additionally, a significant decrease in *Cd11c* expression was observed in the MIA group (Fig. 2E, *p* = 0.035).

### Reduced myelination in the brains of the mice derived from the MIA model

**Fig. 3:**
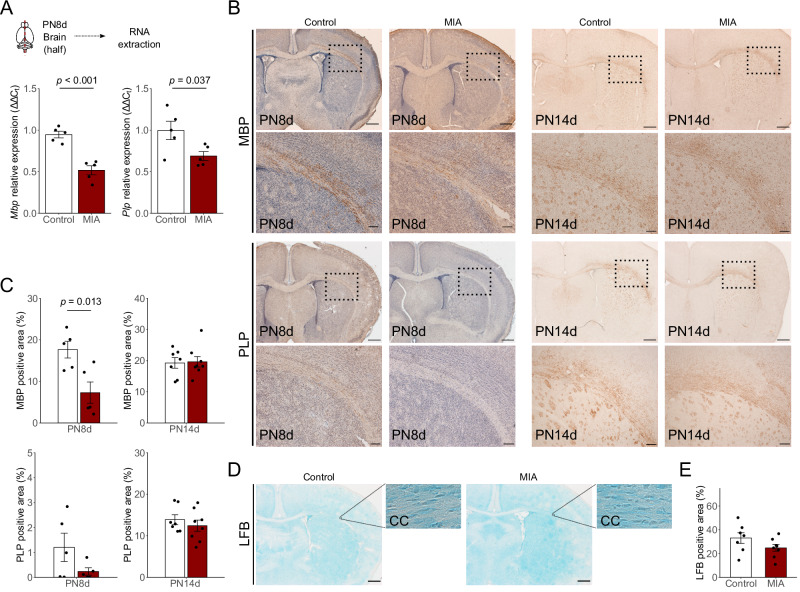
Comparison of myelination-related mRNA and protein. **A** Protocol of RNA extraction. *Mbp* and *Plp* mRNA expression in PN8d offspring between the control and MIA groups (*n* = 5 in each group; maximum 2 offspring per litter). **B** MBP and PLP immunostaining of the lateral corpus callosum sections of the control and MIA groups at PN8d (left panel) and PN14d (right panel). Scale bar = 500 µm (lower magnified image) and 200 µm (higher magnified image). **C** Quantitation results of the percentage of the positive expression area of MBP (upper panel) and PLP (lower panel) in PN8d (*n* = 5 in each group; maximum 2 offspring per litter) and PN14d offspring (*n* = 7 in the control group and *n* = 8 in the MIA group; maximum 2 offspring per litter). **D** Luxol Fast Blue staining of the lateral corpus callosum of the control and MIA groups at PM14d. Scale bar = 500 µm; Magnified images were cropped into 114 × 68 µm. **E** Quantitation results of the percentage of the Luxol Fast Blue positively stained area in PN14d offspring (*n* = 7 in the control group and *n* = 8 in the MIA group; maximum 2 offspring per litter). Data are presented as means ± SEMs. MIA maternal immune activation, MBP myelin basic protein, PLP proteolipid protein, qRT-PCR quantitative reverse transcription-polymerase chain reaction, LFB Luxol Fast Blue, SEM standard error of measurement.

The expression levels of *Mbp*, *Plp* (Fig. 3A), *Mog*, *Mag*, and *Olig2* (Supplementary Fig. 3) were compared between the control and MIA groups at PN8d to determine the effect of MIA on myelination. *Mbp* and *Plp* mRNA levels were significantly lower in the MIA group than in the control group (Fig. 3A, *p* < 0.001 and *p* = 0.037, respectively). *Mog*, *Mag*, and *Olig2* mRNA levels did not significantly differ between the control and MIA groups; however, *Mog* and *Mag* expressions showed a lower trend in the MIA group than in the control group (Supplementary Fig. 3). Immunohistochemistry of the lateral corpus callosum showed that MBP expression was significantly reduced in the MIA group at PN8d (Fig. 3B, C, *p* = 0.013). Moreover, PLP expression tended to be reduced in the MIA group; however, no significant difference was detected at PN8d (Fig. 3B, C). These reduced trends of MBP and PLP in the MIA group were not detected at PN14d (Fig. 3B, C). In addition, Luxol Fast Blue staining, performed to detect myelination at PN14d, demonstrated a slightly reduced trend in the corpus callosum in the MIA group, without a significant difference (Fig. 3D, E).

### Delayed myelination in human preterm neonates with HCA

**Fig. 4:**
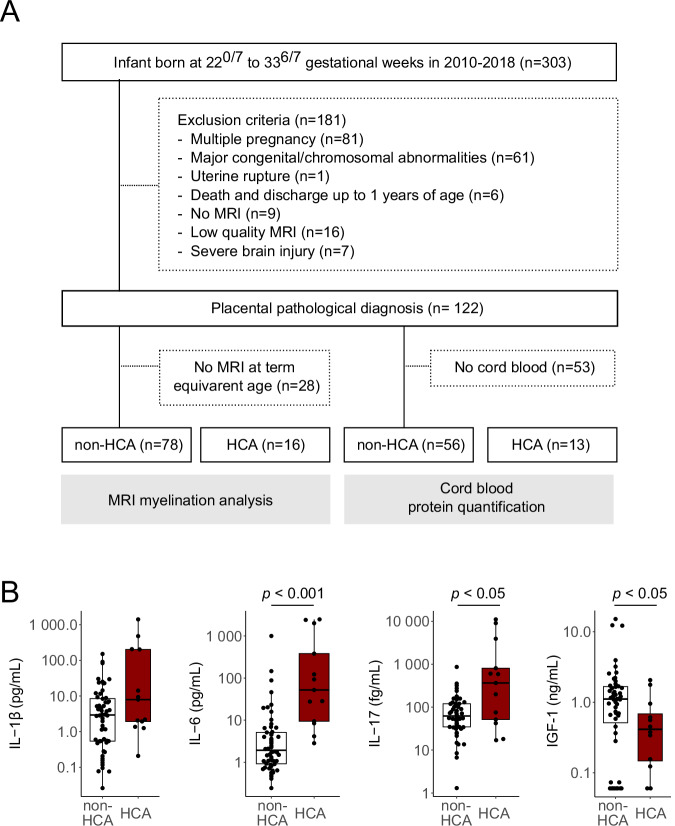
Comparison in human preterm neonates between non-HCA and HCA. **A** Flow chart of study participants. Among 303 individuals, 78 infants without HCA (non-HCA group) and 16 infants with HCA (HCA group) were eligible for MRI evaluation, and 56 infants (non-HCA group) and 13 infants (HCA group) were eligible for cord blood analyses. **B** Levels of pro-inflammatory cytokines and IGF-1 in cord serum samples. Center line of box plot, median; box limits, upper and lower quartiles; whiskers, 1.5x interquartile range; points, outliers. HCA, histological chorioamnionitis.

The incidence of delayed myelination was evaluated in human preterm-born infants using MRI at term-equivalent age. Overall, 122 children born before 34 weeks of gestation were included (Fig. 4A), and the non-HCA and HCA groups were compared. Baseline characteristics of the patients in the MRI myelination analysis are shown in Table 1. Overall, patient characteristics were similar between the groups, except for a significant difference in SGA prevalence, which was higher in the non-HCA group than in the HCA group (*p* = 0.02). Similarly, the z-score of birth weight was significantly lower in the non-HCA group (*p* = 0.048). Neonatal infections were more frequent in the HCA group (*p* = 0.003), and the HCA group exhibited a higher incidence of delayed myelination than the non-HCA group (Table 1, 25.0% vs. 5.1%, *p* = 0.03). Logistic regression analysis revealed that the crude odds ratio for delayed myelination in the HCA group was 6.2 [95% confidential interval: 1.4*–*28.0]. A multivariate analysis was performed with the birth weight z-score and gestational age as covariates to exclude bias caused by birth weight. The odds ratio for delayed myelination in the HCA group remained significantly elevated (adjusted odds ratio 5.9 [1.2–29.4], Table 2).

**Table 1:** Maternal and neonatal characteristics, and delayed myelination in term-equivalent age-MRI between the non-HCA and HCA groups

	non-HCA	HCA	*p*-value
	(*n* = 78)	(*n* = 16)	
Maternal characteristics			
Maternal age (years)	35 [26–42]	37 [25–43]	0.18
Primiparous	41 (52.6%)	6 (37.5%)	0.41^a^
Gestational age at delivery (weeks)	30.8 [24.0–34.0]	31.1 [24.4–33.7]	0.33
Cesarean section	70 (89.7%)	14 (87.5%)	0.68
Antenatal corticosteroid treatment	49 (62.8%)	11 (68.8%)	0.78^a^
Antenatal magnesium sulfate treatment	26 (33.3%)	5 (31.2%)	>0.99
Neonatal characteristics			
Male	44 (58.7%)	8 (50.0%)	0.59^a^
Birth weight (g)	1303 [506–2482]	1358 [624–2294]	0.69
Birth weight (z-score)	−0.60 [−4.10–3.10]	−0.20 [−1.50–1.20]	0.048
Small for gestational age	29 (37.2%)	1 (6.2%)	0.02
Respiratory distress syndrome	40 (51.3%)	7 (43.8%)	0.79^a^
Duration of intubation (days)	1 [0–79]	2 [0–67]	0.94
Bronchopulmonary dysplasia	17 (21.8%)	4 (25.0%)	0.75
Intraventricular hemorrhage (grade 1 or 2)	2 (2.6%)	2 (12.5%)	0.13
Patent ductus arteriosus ligation	1 (1.3%)	0 (0.0%)	>0.99
Inotrope use	5 (6.7%)	3 (18.8%)	0.14
Postnatal steroid use	5 (6.7%)	1 (6.2%)	>0.99
Necrotizing enterocolitis	0 (0.0%)	0 (0.0%)	ND
Infection	3 (3.8%)	5 (31.2%)	0.003
Treated retinopathy of prematurity	7 (9.0%)	2 (12.5%)	0.65
Duration of hospitalization (days)	66 [29–141]	66 [28–156]	0.91
MRI at term-equivalent age			
Delayed myelination	4 (5.1%)	4 (25.0%)	0.03

Continuous variables are presented as mean ± SD or median [minimum–maximum] and *p-*values were calculated by Student’s *t*-test or Mann–Whitney *U* test for normal or non-normal distribution, respectively. Categorical variables are presented as numbers (%) and *p*-values were calculated by Fisher’s exact test.

*HCA* histological chorioamnionitis, *MRI* magnetic resonance imaging, *ND* not detected.

^a^χ^2^ test as appropriate.

**Table 2:** Logistic regression analysis for delayed myelination identified in term-equivalent MRI

	Crude Odds ratio [95% CI]	*p*-value	Adjusted Odds ratio^a^ [95% CI]	*p*-value
HCA	6.2 [1.4–28.0]	0.02	5.9 [1.2–29.4]	0.03

*MRI* magnetic resonance imaging, *CI* Confidence Interval, *HCA* histological chorioamnionitis.

^a^Adjusted for gestational age at delivery (weeks) and birth weight (z-score).

In addition, cord blood levels of IL-6 and IL-17A in the HCA group were significantly higher than those in the non-HCA group (Fig. 4B, *p* < 0.001 and *p* = 0.016, respectively), although the characteristics were similar between the groups (Supplementary Table 1). Similarly, IL-1β levels showed an increasing trend in the HCA group (Fig. 4B, *p* = 0.079). CD11c^+^ microglia are major source of IGF-1 in the developing brain^26^; therefore, IGF-1 concentrations were assessed to indirectly verify whether intrauterine inflammation reduces CD11c^+^ microglia in humans. IGF-1 levels in cord serum in the HCA group were significantly lower than those in the non-HCA group (Fig. 4B, *p* = 0.0495).

### Expression of CD11c in developing brains and patients with ASD via in silico analyses

**Fig. 5:**
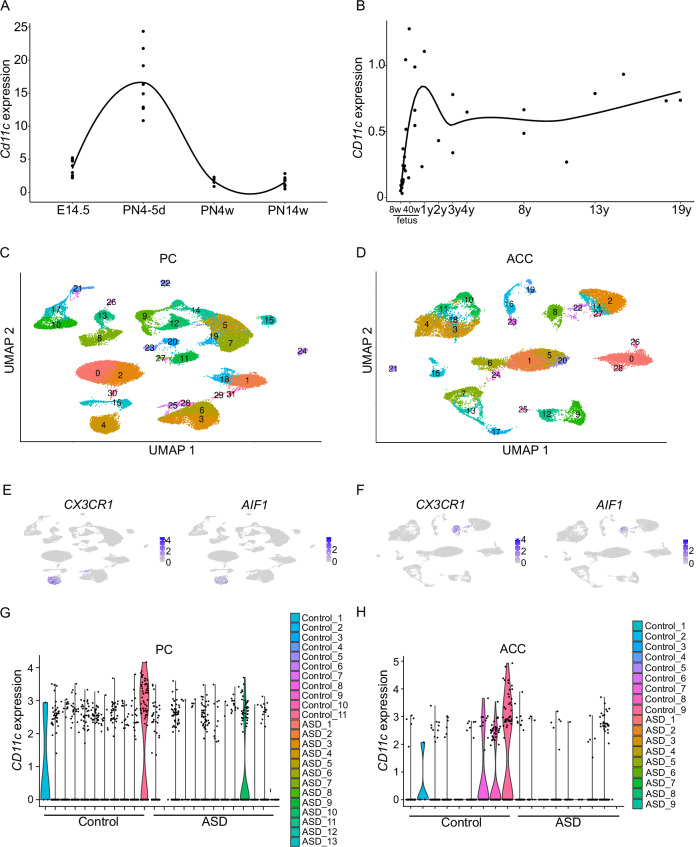
In silico analysis of *CD11c* expression. **A** *Cd11c* expression (count per million) of microglia in the whole mouse brain at each stage of development. Each dot represents the average expression from each sample, and the solid line shows the approximate curve by local polynomial regression fitting. These data were generated using the normalized gene expression dataset from a previous single-cell RNA-seq study [54]. E14.5, PN4–5 d, and PN14w, *n* = 8; PN4w, *n* = 4. **B** *CD11c* expression (reads per kilobase of exon model per million mapped reads) in the human brain at each stage of development. Each dot represents the average expression of each individual integrating multiple sites, and the solid line shows the approximate curve by local polynomial regression fitting. These data were generated using the gene expression dataset from BrainSpan (http://www.brainspan.org/, *n* = 42). **C** Clustering of snRNA-seq data in the PC. **D** Clustering of snRNA-seq data in the ACC. **E** Annotation according to known microglial marker genes in the PC. Microglia were identified as cluster 4. **F** Annotation according to known microglial marker genes in the ACC. Microglia were identified as cluster 8. **G** *CD11c* expression of microglia between control and ASD groups in the PC. **H** *CD11c* expression of microglia between control and ASD groups in the ACC. **C**–**H** Analyses of snRNA-seq data from a previous ASD study [55]. ACC, anterior cingulate cortex; AIF1, Allograft inflammatory factor 1; ASD, autism spectrum disorder; CX3CR1, CX3C motif chemokine receptor 1; PC, prefrontal cortex; UMAP, Uniform Manifold Approximation and Projection.

We evaluated *Cd11c* and *CD11c* expressions during mouse and human neurodevelopment using public data to link results from mouse models and clinical studies on human preterm infants. Their expression peaked (Fig. 5A) on PN4–5 d in mice, was reduced at PN4w, and slightly increased at PN14w. Peak expression in humans occurred from birth until 1 year of age (Fig. 5B). Subsequently, the expression decreased from 2 to 11 years and slightly increased from 13 to 19 years. The overall expression pattern in humans closely resembled that observed in mice, although differences were observed in the length of time owing to differences in lifespan.

We investigated a previous snRNA-seq dataset of children with ASD and controls and identified microglial clusters using annotation genes to compare *CD11c* expression in microglia between patients with ASD and typically developing controls (Fig. 5C–F). A high population of microglia in the PC regions in one child in the ASD group (*n* = 13) and two children in the control group (*n* = 11) expressed *CD11c* (≥25%) (Fig. 5G). However, in the ACC regions, a high population of microglia was deficient in *CD11c* in the ASD group (*n* = 9); nevertheless, its expression was detected in four children in the control group (*n* = 9, Fig. 5H).

## Discussion

This study demonstrated the poor induction of CD11c^+^ microglia at PN3d in an LPS-induced MIA model. Myelination-related protein expression was reduced in the MIA group at PN8d, and those reductions were not detected at PN14d. Furthermore, human preterm infants with HCA showed increased levels of pro-inflammatory cytokines IL-17A and IL-6 and decreased IGF-1 levels at birth. Infants in the HCA group showed an increased trend in the incidence of delayed myelination at term-equivalent age in MRI. Moreover, *CD11c* expression in microglia was transiently induced in human infants within the first year of life.

A previous study reported that the proportion of CD11c^+^ microglia increases at PN3d but decreases at PN7d^26^, consistent with our control group results. Similarly, a recent region-specific study demonstrated that CD11c^+^ microglia were reduced at PN7d compared to PN4d in most brain regions, except for the cerebellum^38^. In silico analysis also showed that *Cd11c* expression was transiently induced within PN4–5 d in murine microglia. A similar induction in microglia was observed in human infants under one year of age. Thus, transient induction of CD11c^+^ microglia after birth may be significant in human neurodevelopment.

A high cell population of CD11c^+^ microglia was not detected in microglia of the ACC regions among children with ASD; however, this was detected in some of the control children. This poor induction of CD11c^+^ microglia is consistent with a previous report showing reduced CD11c^+^ microglia in patients with ASD using random sampling from postmortem brains^39^. Moreover, *CD11c* expression was reduced in the postmortem dorsolateral prefrontal cortex of patients with high-inflammatory schizophrenia^40^, which shares features with ASD. Thus, poor *CD11c* expression in microglia may be related to ASD pathogenesis.

The function of CD11c^+^ microglia remains controversial, and the definitions of “good” or “bad” microglia are currently being reassessed owing to recent insights into the vast repertoire of microglia and their plasticity^41^. The CD11c protein was initially increased in disease-associated microglia (DAM)^42^; however, its expression did not correlate with the degree of neuronal cell loss in adulthood^43^. CD11c^+^ microglia contribute to white matter repair, oligodendrocyte maturation, and functional recovery after ischemic stroke^44^; nevertheless, their function remains not fully understood^45^.

Axon tract-associated microglia (ATM), which express high levels of SPP1 and LGALS1, appear during narrow windows in early development^46^. SPP1 and LGALS1 support oligodendrocytes and promote axonal growth, respectively, and are expressed in CD11c^+^ microglia^26^. Furthermore, CD11c^+^ microglia produce IGF-1. Thus, CD11c^+^ microglia are consistent with ATM. PAM appears in the developing corpus callosum and cerebellar white matter during a short period in the first postnatal week^27^. These microglia express *Spp1*, *Cd11c*, and *Igf1*, which share a transcriptional signature with DAM. This is likely the cause of the confusion. Considering the heterogeneity of microglia, CD11c^+^ microglia have been recognized as a new type involved in developmental neurogenesis and myelination^47^, a notion supported by our findings.

Expressions of myelination-related proteins, including *Plp* and *Mbp*, were significantly reduced in the MIA group at PN8d, following the poor induction of CD11c^+^ microglia at PN3d. In addition, a reduced number of oligodendrocytes, key players in axonal myelination, was observed in pups from our previous MIA model^22^. CD11c^+^ microglia are involved in the maturation of oligodendrocyte precursors via IGF-1 secretion^26^. These findings suggest that induction inhibition of CD11c^+^ microglia at PN3d may reduce myelination by inhibiting the maturation of oligodendrocyte precursors in the MIA group. Developing methods for increasing the number of CD11c^+^ microglia in the MIA group is necessary to establish a causal relationship between the reduction of CD11c^+^ microglia and impaired myelination in MIA.

Notably, reduced myelination has been observed in children with ASD^48^, and ASD-like behavioral deficits in LPS-induced MIA models were demonstrated in our previous report^22^. Reduced myelination or decreased myelination-related proteins play a role in ASD pathophysiology^49–54^. Similarly, reduced myelination and oligodendrocyte-related gene expression have been observed in poly(I:C)-induced MIA models, which exhibit an ASD-like phenotype^55^, consistent with the results of our LPS-induced MIA models. Thus, reduced myelination may explain a part of the ASD-like behavioral impairments detected in the LPS-induced MIA model.

In this study, delayed myelination at term-equivalent age was observed in human preterm infants exposed to HCA, consistent with previous findings showing that HCA increases the risk of brain injury and delayed maturation in preterm infants based on MRI data acquired at term-equivalent age^56^. Moreover, reduced expression of myelination-related proteins was detected in the MIA model at PN8d, corresponding to human-term gestation. A recent study reported that delayed myelination at term-equivalent age was associated with poor short-term prognosis^57^. This may suggest that adequate myelination at the appropriate developmental stage has important implications for neurodevelopment, although further studies are required to confirm this. In addition, pro-inflammatory cytokines, including IL-6 and IL-17A, were significantly increased in the cord serum of preterm neonates exposed to HCA compared to those without HCA, in line with the finding that Th17-type responses are amplified in preterm neonates exposed to HCA^58^. Notably, we previously reported that IL-6 and IL-17A were increased in the pups of this MIA model^21,59^. These findings suggest that our MIA model mimics HCA-related brain injury. Thus, poor induction of CD11c^+^ microglia detected in our MIA model may occur in HCA-related brain injury.

RNA-seq analysis revealed significant differences in the overall gene expression signature of microglia at PN3d in the LPS-induced MIA model compared to the control group. Among the altered pathways, the inflammatory response pathway ranked third. In contrast to *Cd11c*, *Lgals3* and *Il1b* expressions were increased in the MIA group. Therefore, these microglia, which highly expressed *Lgals3*, *Il1b*, and other proinflammatory cytokines in response to LPS, may differ from the CD11c^+^ microglia or ATM populations, although *Lgals3* is an ATM marker. Previous reports indicated that LPS elevates *Lgals3*^60^, *Il1b*, and *Ccl3*^61^ expression in microglia but does not upregulate CD11c expression^62^, consistent with our findings. We believe that these microglia expressing *Lgals3* and *Il1b* play a pathological role in addition to the decreased CD11c^+^ microglia because IL-1β is known to be associated with the pathogenesis of neuroinflammation in MIA^34^.

We observed an interesting phenomenon regarding the number of neutrophils in the developing brain. An initial increase was observed in the number of neutrophils in the PN1d brain, which subsequently decreased to the same level as the control group in PN3d. Neutrophils are involved in microglial activation during hypoxia-ischemia insults in the neonatal brain^63,64^. In the context of the LPS-induced MIA model, the induction of neutrophils appears to contribute to microglia activation, further highlighting the complex interplay between different immune cell populations.

To the best of our knowledge, our study provided the first evidence of the failure to induce CD11c^+^ microglia in an LPS-induced MIA model, demonstrated using flow cytometry, immunofluorescence imaging, and mRNA analysis. However, this study had several limitations. First, we could not directly investigate whether CD11c expression had changed in the microglia of human neonates born to mothers with HCA, despite observing an increase in IL-17A and a decrease in IGF-1. Instead, we observed that *CD11c* levels in microglia were reduced in ASD brains. Second, the HCA group had a significantly lower frequency of infants with SGA, which could introduce bias. However, the association between SGA and delayed myelination remains not fully understood. SGA is recognized as a risk factor for cognitive impairment^65^ and may exhibit reduced myelination^66^. Considering these findings, a higher incidence of delayed myelination in the HCA group is likely reliable. Moreover, after adjusting for birth weight, multivariate analysis revealed a significantly higher incidence of delayed myelination in the HCA group. However, our data were insufficient to compare long-term prognoses. Further studies in larger populations are necessary to confirm the results of the present study. Third, the present study could not show whether IGF-1 expression would be altered in CD11c^+^ microglia of the MIA group at PN3d, although a previous study reported IGF-1 expression in CD11c^+^ microglia^26^. Further studies are required to confirm this. Moreover, preparing adequate numbers of mice to investigate sex differences was difficult owing to the low survival rates of pups after LPS administration. The study results do not reveal detailed associations with sex; nevertheless, the results may be considered common to both sexes. Finally, further investigations of the therapeutic effect of CD11c^+^ microglia in the offspring of the MIA model are necessary, even though their effectiveness has been reported in the context of stroke^44^. Further research is required to evaluate sex differences and the therapeutic effects of CD11c^+^ microglia in the specific context of MIA-related neurodevelopmental disorders, such as ASD.

In summary, prenatal inflammation resulted in poor induction of CD11c^+^ microglia during the early developmental window, which may be related to myelination inhibition during the subsequent stage. This process may underlie the impaired neurodevelopment in preterm infants with HCA, although further investigations are required.

## Methods

### Animals and treatments

We have complied with all relevant ethical regulations for animal use. The animal protocols used in this study were approved by the Animal Experiment Committee of Nagoya University (approval number: M220211-001). All pregnant Slc:ICR (CD-1) mice (8*–*9 weeks; Japan SLC, Shizuoka, Japan) were maintained under a standard specific pathogen-free environment with a 12 h light/dark cycle (9:00–21:00) and were provided free access to food and water. The pregnant mice were randomly assigned to the control and MIA groups, as in our previous reports^21,22^. Briefly, dams in the MIA group were intraperitoneally injected with 50 μg LPS (serotype O55: B5; Sigma-Aldrich, St. Louis, MO, USA) dissolved in 500 μL phosphate-buffered saline (PBS) on gestational day 17. This dose corresponded to approximately 0.77 mg/kg LPS. Dams in the control group received an equal volume of PBS (Fig. 1A). Offspring were randomly collected from each litter without determining their sex and were deeply anesthetized by icing (for PN1–3 d) or isoflurane with the open drop method (for PN8d or more) for subsequent analyses. For each experiment, 3–9 offspring from 2–6 litters were used.

### Flow cytometry analysis of microglia and immune cells from MIA offspring

The procedures were performed as in previous studies^33,67^. Briefly, PN1d, 3 d, 8 d, and postnatal week 6 (PN6w) offspring were transcardially perfused with PBS (*n* = 3–9 from 2 to 6 litters). Their brains were individually dissected, minced into 1 mm^3^ pieces in Neural Tissue Dissociation Kit – Postnatal Neurons (Miltenyi Biotec, Bergisch-Gladbach, Germany) or collagenase buffer, and incubated at 37 °C for 15 min in a gentle MACS Dissociator (Miltenyi Biotec). The cells were re-suspended in 37% Percoll (Sigma-Aldrich) and centrifuged at 760 × *g* for 20 min to remove myelin debris. The cells containing microglia and immune cells were incubated with anti-CD16/CD32 antibodies (Thermo Fisher Scientific, Waltham, MA, USA) to block Fc receptors and were subsequently stained with fluorescence-conjugated monoclonal antibodies for CD11b (Cat# 557657), CD45 (Cat# 552848), CD11c (Cat# 550261), CD3e (Cat# 551163), Ly-6G (Cat# 551461), and Ly-6C (Cat# 553104), all of which were purchased from BD Biosciences (Franklin Lakes, NJ, USA). Flow cytometry was performed with FACSVerse Flow Cytometer (BD Biosciences), and the data were analyzed using FlowJo Software (BD Biosciences). The cell types were differentiated using surface markers (Fig. 1B): CD45^high^CD11b^−^CD3e^+^ as T cells, CD45^high^CD11b^+^Ly-6C^+^Ly-6G^−^ as monocytes, CD45^high^CD11b^+^Ly-6C^+^Ly-6G^+^ as neutrophils, and CD45^low^CD11b^+^ as microglia.

### Total RNA extraction from microglia and the whole brain of MIA offspring

The PBS-perfused brain tissues of PN3d offspring were dissociated into single cells using the same abovementioned method for RNA analysis of microglia. Microglia were isolated from dissociated cells using magnetic-activated cell sorting (MACS) to minimize cytotoxic effects on RNA analysis, following previous reports^33,43,67^. Briefly, myelin debris was removed using Myelin Removal Beads II (Miltenyi Biotec), and purified cells were incubated with anti-CD16/CD32 antibodies (Thermo Fisher Scientific, Waltham, MA, USA) to block Fc receptors, followed by an incubation with anti-CD11b MicroBeads (Miltenyi Biotec) to isolate microglia through autoMACS Columns (Miltenyi Biotec). Total RNA was extracted from MACS-isolated microglia using an RNeasy Micro Kit (Qiagen, Hilden, Germany), according to the manufacturer’s instructions.

PBS-perfused hemispheres from PN8d offspring were dissected and homogenized in QIAzol (Qiagen) for myelination-related RNA analysis. Total RNA was extracted using an RNeasy mini kit (Qiagen), according to the manufacturer’s instructions.

### RNA-sequence (seq) analysis of microglia from MIA offspring

Total RNA of microglia from PN3d offspring in the control and MIA groups (*n* = 3 from two litters in each group) were qualified using an Agilent 2100 Bioanalyzer (Agilent Technologies, Santa Clara, CA, USA), and libraries were prepared using TruSeq stranded mRNA Library Prep (Illumina, San Diego, CA, USA). From these libraries, 150 bp paired-end reads were sequenced on a HiSeq X Ten system using HiSeq X Reagent Kits (Illumina) and a NovaSeq 6000 system using NovaSeq Reagent Kits (Illumina). For RNA-seq data analysis, reference genome assemblies and gene annotations were retrieved from iGenomes (https://sugenomeslumina.com/sequencing/sequencing_software/igenome.html) using the mouse genome version University of California Santa Cruz (UCSC) mm10. The Illumina adapter sequences and low-quality bases (quality score < 20) were trimmed from the 3′ ends of the sequencing reads using Trim Galore v.0.5.0 (https://www.bioinformatics.babraham.ac.uk/projects/trim_galore/). The reads were qualified before and after trimming using FastQC v.0.11.8 (https://www.bioinformatics.babraham.ac.uk/projects/fastqc/). Thereafter, processed reads were mapped on the reference genome assembly using HISAT2 v2.1.0^68^. Annotated gene expressions from mapping data were quantified using StringTie v1.3.5^69^. Differential expression analysis was performed using edgeR v3.24.3^70^ implemented in R v3.5.1, and multiple testing correction for the *p*-values was performed using the qvalue package v2.24.1^71^ in R. A heatmap of differentially expressed genes (DEGs: |log_2_FC | > 1.25 and *p* < 0.05) was constructed using the heatmap3 package in RStudio with Ward’s method (RStudio version 2022.07.2 + 576 and R version 4.2.1). We employed iDEP (integrated Differential Expression and Pathway analysis, version 1.0) software (http://bioinformatics.sdstate.edu/idepg/) for volcano plot analysis^72^. Moreover, we utilized Metascape for gene ontology (GO) enrichment analysis (http://metascape.org).

### Quantitative reverse transcription-polymerase chain reaction (qRT-PCR)

Complementary DNA (cDNA) was generated using 100 ng of total RNA through first-strand cDNA synthesis (ReverTra Ace; Toyobo Co., Ltd., Osaka, Japan). Gene expression was evaluated using Thermal Cycler Dice (Takara Bio Inc., Tokyo, Japan) and SYBRII Premix Ex Taq (Takara Bio Inc.) reagents. The expression levels of *Ccl3, Cxcl1, Cxcl10, Il1b, Lgals3*, and *Cd11c* were examined in MACS-isolated microglia samples of PN3d offspring in the control and MIA groups (*n* = 6 in each group; maximum two offspring per litter). In addition, *Mbp*, *Plp*, *Mog*, *Mag*, and *Olig2* expression levels were examined in whole-brain samples of PN8d offspring (*n* = 5 in each group; maximum two offspring per litter). The expression level of *Actb* served as an endogenous control. The primers used for qRT-PCR are listed in Supplementary Table 2.

### Immunohistochemistry

We evaluated early changes in the myelination-related proteins in PN8d and PN14d MIA offspring, corresponding to the human-term gestation and 1–2 years postnatal, respectively^73^. PN8d and PN14d offspring (*n* = 5–8 in each group; maximum two offspring per litter) were transcardially perfused with PBS, followed by 4% paraformaldehyde (PFA). Dissected brains were fixed in 4% PFA overnight at 4 °C and subsequently cryoprotected with 30% sucrose at 4 °C. Tissues were embedded in optimal cutting temperature (OCT) compound (Tissue-Tek) and were sectioned using a cryostat (Leica Biosystems, Nussloch, Germany). Free-floating coronal sections (50 µm thickness, Bregma +0.5 mm in Allen Brain Atlas [https://mouse.brain-map.org/static/atlas]) were stored in PBS. Sections were blocked using goat serum for 10 min and were subsequently incubated with the primary antibodies overnight at 4 °C. The following primary antibodies were used: rabbit monoclonal anti-PLP (myelin proteolipid protein, 1:200, #ab105784; Abcam plc, Cambridge, UK) and rabbit polyclonal anti-MBP (myelin basic protein, 1:200, #AB980; Millipore, Temecula, CA, USA). Next, sections were incubated with a biotinylated secondary antibody (Nichirei Bioscience Inc.) for 10 min at room temperature and visualized using diaminobenzidine (DAB; Agilent Technologies). Images were acquired using the AXIO Examiner (Carl Zeiss AC, Jena, Germany) at ×10 magnification. Images were cropped into rectangles (454 × 272 µm) and analyzed using color deconvolution and the particle analysis plug-in of the Fiji platform Version 2.3 (ImageJ distribution, http://fiji.sc/Fiji) to quantify the DAB staining area at the lateral part of the corpus callosum.

### Immunofluorescence staining

PN3d and PN8d offspring (*n* = 6 from 2 to 3 litters in each group) were transcardially perfused with PBS, followed by 10–15 mL of Zamboni solution (Wako, Osaka, Japan). Subsequently, the dissected brains were fixed in Zamboni solution overnight at 4 °C and then cryoprotected with 30% sucrose at 4 °C. Tissues were embedded in OCT compound (Tissue-Tek) and were sectioned using a cryostat (Leica Biosystems). Free-floating coronal sections (50 µm) of the corpus callosum (Bregma +0.5 mm in Allen Brain Atlas) and hippocampus (Bregma −1.6 mm in Allen Brain Atlas) were stored in PBS. Sections were blocked and permeabilized with 5% goat serum and 0.3% Triton-X in PBS for 30 min at room temperature and subsequently incubated with the primary antibody for 48 h at 4 °C, followed by incubation with the secondary antibody and DAPI staining for 2 h at room temperature. Sections were washed, transferred onto glass slides, and mounted in Dako mounting medium (Agilent). Images were acquired using confocal microscopy (Elyra PS.1, Carl Zeiss AC), and the cell number was quantified using the Fiji platform Version 2.3.

The following primary antibodies were used: rabbit anti-Iba1 (1:500, #019-19741; Wako); Armenian hamster anti-CD11c (1:10, #550283; BD Biosciences); and rat anti-CLEC7A (1:50, #mabg-mdect-2; InvivoGen, San Diego, CA, USA). The following secondary antibodies were used: goat anti-rabbit Alexa568 (1:500, #A11036; Invitrogen); goat anti-Hamster DyLight488 (1:500, #405503, BioLegend); and goat anti-rat Alexa647 (1:500, #A21247; Invitrogen).

### Luxol fast blue staining

We examined the PN14d section (*n* = 7–8 offspring from 4 litters in each group) since myelination is reportedly not detected by Luxol Fast Blue at PN8d^74^. Sections on slides were placed into a staining solution of 0.1% Luxol Fast Blue (Sigma-Aldrich) for 2 h at 60 °C. Sections were washed in 95% ethanol and water and immersed in 0.2% lithium carbonate, and staining was differentiated in 70% ethanol.

### Evaluation of myelination in preterm infants exposed to in-utero inflammation

We examined MRI images of infants diagnosed with or without HCA and compared the incidence of delayed myelination to assess the impact of in-utero inflammation on myelination. Delayed myelination has been suggested to be associated with developmental delay^75^ and ASD^48^. Furthermore, an MRI scoring system at term-equivalent age, which included delayed myelination, was inversely correlated with motor, memory, and learning outcomes at ages two and seven^76,77^. Furthermore, delayed myelination at term-equivalent age was independently associated with the General Quotient (GQ), Personal-Social sub-quotient (PS), and Eye-Hand Coordination sub-quotient (EHC) of the Griffiths Mental Development Scales at 6–24 months of age^57^.

This retrospective cohort study included preterm infants born at Nagoya University Hospital from January 2010 to December 2018. The inclusion criteria were preterm infants between 22^0/7^ and 33^6/7^ gestational weeks admitted to the neonatal intensive care unit. The exclusion criteria were multiple pregnancies, major congenital and/or chromosomal abnormalities, severe brain injury, and missing data. Data on maternal and neonatal characteristics were collected from electronic medical records^78,79^. Maternal characteristics included maternal age, parity, gestational age at delivery, cesarean section, antenatal corticosteroid treatment history, and magnesium treatment history. Neonatal characteristics included sex, birth weight, small-for-gestational-age (SGA) status, respiratory distress syndrome, duration of intubation, bronchopulmonary dysplasia, intraventricular hemorrhage (grade 1 or 2), patent ductus arteriosus ligation, inotrope use, postnatal steroid use, necrotizing enterocolitis, infection, treatment for retinopathy of prematurity, and hospitalization duration.

HCA was diagnosed using the Blanc classification^80^. Patients with stage 2 (moderate) and 3 (severe) were included in the HCA group, whereas those with no chorioamnionitis or stage 1 (mild) were included in the non-HCA group^56^. MRI was performed in infants when they reached term-equivalent age (37^0/7^–41^6/7^ weeks after conception) using a 3.0 Tesla scanner system (MAGNETOM Verio or TrioTim; Siemens, Erlangen, Germany). MRI assessment of delayed myelination was performed by an experienced pediatric neurologist (H.K.), who was blinded to any clinical and research data. The evaluation followed the methodology outlined in a previous study^81^.

### Assessment of inflammatory cytokines and IGF-1 concentration in umbilical cord serum at birth

Umbilical cord blood samples were collected at delivery, and serum was separated by centrifugation at 1700 × *g* for 10 min and stored at −80 °C, as previously reported^82^. Cord sample concentrations of IL-17A (S-PLEX Human IL-17A Kit; Meso Scale Diagnostics, LLC., Rockville, MD, USA), IL-6, and IL-1β (V-PLEX Custom Human Cytokine; Meso Scale Diagnostics, LLC.) were measured using a MESO QuickPlex SQ 120 system (Meso Scale Diagnostics, LLC.). The concentrations of IGF-1 were measured using an enzyme-linked immunosorbent assay kit (#DG100B; R&D Systems, Minneapolis, MN, USA) according to the manufacturer’s instructions.

### In silico analysis

The RNA seq-based mRNA profile datasets of microglia isolated from mice and bulk brain tissues from humans during neurodevelopment were downloaded from a previous report^46^ and the ‘BrainSpan: Atlas of the Developing Human Brain’ (Allen Institute for Brain Science, data accessed on April 5, 2023, from http://www.brainspan.org/), respectively, to investigate variations in the expressions of *Cd11c* and *CD11c* throughout the developmental period in mice and humans. Single-nucleus (sn) RNA-seq data from postmortem brain samples were downloaded from the National Center for Biotechnology Information Sequence Read Archive database (accession number PRJNA434002)^83^ using the parallel-fastq-dump package (ver. 0.6.7) (https://github.com/rvalieris/parallel-fastq-dump) to investigate *CD11c* expression in patients with ASD. This snRNA-seq data was originally generated from the prefrontal cortex (PC) and anterior cingulate cortex (ACC) of 15 children with ASD (12 males, three females; mean age, 14.8 years) and 16 typically developing children serving as controls (12 males, four females; mean age, 13.4 years). The generated fastq files were aligned and quantified to the GRCh38 human reference genome using 10x Genomics Cell Ranger 7.1.0^84^. Downstream analysis was performed using the Seurat package (ver. 4.3.0)^85^ for R software (ver. 4.2.2). Nuclei expressing <400 genes or >6000 genes were filtered, and nuclei with mitochondrial gene expression exceeding 1% were also excluded to ensure the quality of the nuclei used in the downstream analyses. Consequently, 70,593 nuclei from 24 PC samples and 47,315 nuclei from 18 ACC samples were retrieved. Next, the snRNA-seq matrix was normalized using the LogNormalize function and was scaled and centered using the ScaleData function of the Seurat package. Batch effect correction was performed using the Harmony package (ver. 0.1.1)^86^. Finally, the data were visualized using Uniform Manifold Approximation and Projection to reduce dimensionality and enable nuclei visualization in a two-dimensional plot.

### Statistics and reproducibility

The variables are presented as means ± standard errors of measurement (SEMs) and were evaluated using the Student’s *t-*test for a normal distribution. The median [minimum–maximum] was calculated for non-normally distributed variables, and the Mann–Whitney *U* test was used for comparison. Categorical variables were reported as numbers (%) and compared using Fisher’s exact test or χ^2^ test, as appropriate. All statistical tests were two-sided, and statistical significance was set at *p* < 0.05. All statistical analyses were performed using IBM SPSS Statistics for Windows, Version 28.0 (IBM Corp., Armonk, NY, USA) or Easy R version 1.54 (Saitama Medical Center, Jichi Medical University, Saitama, Japan), a modified version of R and R Commander^87^.

In animal experiments, each analysis utilized independent offspring from each group (*n* = 3–9) as separate individuals. The maximum number of offspring used from a single litter is detailed in the figure legends.

### Ethics and inclusion statement

This study was conducted in collaboration with local researchers, who contributed to the study’s design, implementation, and analysis. All collaborators meeting the authorship criteria have been included as co-authors, with other contributions acknowledged appropriately. Roles and responsibilities were agreed upon in advance, and local relevance was ensured through active consultation with regional partners.

All ethical regulations relevant to human research participants were followed, with approval obtained from the Institutional Ethics Committee of Nagoya University Hospital (approval numbers: 2015-0068 and 2018–0026). Written informed consent was obtained from the parents of all neonates. Measures were taken to minimize risks to participants, avoiding stigmatization or harm, and local studies relevant to the findings have been appropriately cited.

### Glossary

MIA, Maternal Immune Activation. HCA, Histological Chorioamnionitis. PN3d, Postnatal Day 3. IL-6, Interleukin-6. IL-17A, Interleukin-17A. IGF-1, Insulin-like Growth Factor 1. ASD, Autism Spectrum Disorder. MRI, Magnetic Resonance Imaging. PBS, Phosphate-Buffered Saline. LPS, Lipopolysaccharide. Th17, T-helper 17. T cells, T-lymphocytes. RNA-seq, RNA sequencing. qRT-PCR, Quantitative Reverse Transcription Polymerase Chain Reaction. OCT, Optimal Cutting Temperature. PLP, Myelin Proteolipid Protein. MBP, Myelin Basic Protein. DAB, Diaminobenzidine. CLEC7A, C-type Lectin Domain Family 7 Member A. PAM, Proliferative Region-Associated Microglia. ACC, Anterior Cingulate Cortex. PC, Prefrontal Cortex. DAM, Disease-Associated Microglia. ATM, Axon Tract-Associated Microglia.

### Reporting summary

Further information on research design is available in the Nature Portfolio Reporting Summary linked to this article.

## Supplementary information

Transparent Peer Review file

Supplementary information

Description of Additional Supplementary Materials

Supplementary Data 1

Reporting Summary

## Data Availability

The RNA-seq data have been deposited with links to BioProject accession number PRJDB15535 in the DDBJ BioProject database. All data supporting the findings of this study are available within the paper and its Supplementary Data. Other data supporting the findings of this study are available from the corresponding author (TK) upon reasonable request.

## Code availability

This study did not use any custom code or mathematical algorithms central to its conclusions.

## References

[1] Estes, M. L. & McAllister, A. K. Maternal immune activation: Implications for neuropsychiatric disorders. *Science* **353**, 772–777 (2016). 27540164 10.1126/science.aag3194 PMC5650490

[2] Knuesel, I. et al. Maternal immune activation and abnormal brain development across CNS disorders. *Nat. Rev. Neurol.* **10**, 643–660 (2014). 25311587 10.1038/nrneurol.2014.187

[3] Choi, G. B. et al. The maternal interleukin-17a pathway in mice promotes autism-like phenotypes in offspring. *Science* **351**, 933–939 (2016). 26822608 10.1126/science.aad0314 PMC4782964

[4] Kim, S. et al. Maternal gut bacteria promote neurodevelopmental abnormalities in mouse offspring. *Nature* **549**, 528–532 (2017). 28902840 10.1038/nature23910 PMC5870873

[5] Fujitani, M., Miyajima, H., Otani, Y. & Liu, X. Maternal and adult Interleukin-17A exposure and autism spectrum disorder. *Front. Psychiatry* **13**, 836181 (2022). 35211045 10.3389/fpsyt.2022.836181 PMC8861354

[6] Thawley, A. J. et al. Aberrant IL-17 levels in rodent models of autism spectrum disorder: a systematic review. *Front. Immunol.* **13**, 874064 (2022). 35757754 10.3389/fimmu.2022.874064 PMC9226456

[7] Wong, H. & Hoeffer, C. Maternal IL-17A in autism. *Exp. Neurol.* **299**, 228–240 (2018). 28455196 10.1016/j.expneurol.2017.04.010 PMC5656543

[8] Lawrence, S. M. & Wynn, J. L. Chorioamnionitis, IL-17A, and fetal origins of neurologic disease. *Am. J. Reprod. Immunol.* **79**, e12803 (2018). 29271527 10.1111/aji.12803 PMC5966827

[9] Paz-Levy, D. et al. Inflammatory and vascular placental lesions are associated with neonatal amplitude integrated EEG recording in early premature neonates. *PLoS ONE* **12**, e0179481 (2017). 28644831 10.1371/journal.pone.0179481 PMC5482430

[10] Shi, Z. et al. Chorioamnionitis in the development of cerebral palsy: a meta-analysis and systematic review. *Pediatrics*. **139**, e20163781 (2017). 10.1542/peds.2016-3781 PMC5470507 28814548

[11] van Vliet, E. O. et al. Placental pathology and long-term neurodevelopment of very preterm infants. *Am. J. Obstet. Gynecol.* **206**, 489.e481–487 (2012). 10.1016/j.ajog.2012.03.024 22521456

[12] Venkatesh, K. K. et al. Histologic chorioamnionitis and risk of neurodevelopmental impairment at age 10 years among extremely preterm infants born before 28 weeks of gestation. *Am. J. Obstet. Gynecol.* **223**, 745.e741–745.e710 (2020). 10.1016/j.ajog.2020.05.001 PMC7609587 32387324

[13] Ylijoki, M. K., Ekholm, E., Ekblad, M. & Lehtonen, L. Prenatal risk factors for adverse developmental outcome in preterm infants-systematic review. *Front. Psychol.* **10**, 595 (2019). 30971974 10.3389/fpsyg.2019.00595 PMC6445261

[14] Raghavan, R. et al. Preterm birth subtypes, placental pathology findings, and risk of neurodevelopmental disabilities during childhood. *Placenta* **83**, 17–25 (2019). 31477202 10.1016/j.placenta.2019.06.374 PMC6727987

[15] Favrais, G. et al. Partial protective effects of melatonin on developing brain in a rat model of chorioamnionitis. *Sci. Rep.* **11**, 22167 (2021). 34773065 10.1038/s41598-021-01746-w PMC8589852

[16] Lei, J. et al. Maternal glucose supplementation in a murine model of chorioamnionitis alleviates dysregulation of autophagy in fetal brain. *Reprod. Sci.* **25**, 1175–1185 (2018). 29017418 10.1177/1933719117734321 PMC6346301

[17] Brosius Lutz, A., Al-Nasiry, S., Kramer, B. W. & Mueller, M. Understanding host-pathogen interactions in acute chorioamnionitis through the use of animal models. *Front. Cell Infect. Microbiol.* **11**, 709309 (2021). 34386434 10.3389/fcimb.2021.709309 PMC8353249

[18] Burd, I., Brown, A., Gonzalez, J. M., Chai, J. & Elovitz, M. A. A mouse model of term chorioamnionitis: unraveling causes of adverse neurological outcomes. *Reprod. Sci.* **18**, 900–907 (2011). 21421895 10.1177/1933719111398498 PMC3343123

[19] Elovitz, M. A. et al. Intrauterine inflammation, insufficient to induce parturition, still evokes fetal and neonatal brain injury. *Int. J. Dev. Neurosci.* **29**, 663–671 (2011). 21382466 10.1016/j.ijdevneu.2011.02.011 PMC3140629

[20] Gan, X. W. et al. De novo Synthesis of SAA1 in the placenta participates in parturition. *Front. Immunol.* **11**, 1038 (2020). 32582166 10.3389/fimmu.2020.01038 PMC7297131

[21] Imai, K. et al. Neuroprotective potential of molecular hydrogen against perinatal brain injury via suppression of activated microglia. *Free Radic. Biol. Med.* **91**, 154–163 (2016). 26709014 10.1016/j.freeradbiomed.2015.12.015

[22] Imai, K. et al. Administration of molecular hydrogen during pregnancy improves behavioral abnormalities of offspring in a maternal immune activation model. *Sci. Rep.* **8**, 9221 (2018). 29907804 10.1038/s41598-018-27626-4 PMC6003913

[23] Rigby, M. J., Gomez, T. M. & Puglielli, L. Glial cell-axonal growth cone interactions in neurodevelopment and regeneration. *Front. Neurosci.* **14**, 203 (2020). 32210757 10.3389/fnins.2020.00203 PMC7076157

[24] Reemst, K., Noctor, S. C., Lucassen, P. J. & Hol, E. M. The indispensable roles of microglia and astrocytes during brain development. *Front. Hum. Neurosci.* **10**, 566 (2016). 27877121 10.3389/fnhum.2016.00566 PMC5099170

[25] Wlodarczyk, A. et al. Pathologic and protective roles for microglial subsets and bone marrow- and blood-derived myeloid cells in central nervous system inflammation. *Front. Immunol.* **6**, 463 (2015). 26441968 10.3389/fimmu.2015.00463 PMC4562247

[26] Wlodarczyk, A. et al. A novel microglial subset plays a key role in myelinogenesis in developing brain. *Embo J.* **36**, 3292–3308 (2017). 28963396 10.15252/embj.201696056 PMC5686552

[27] Li, Q. et al. Developmental heterogeneity of microglia and brain myeloid cells revealed by deep single-cell RNA sequencing. *Neuron* **101**, 207–223.e210 (2019). 10.1016/j.neuron.2018.12.006 PMC6336504 30606613

[28] Kohno, K. et al. A spinal microglia population involved in remitting and relapsing neuropathic pain. *Science* **376**, 86–90 (2022). 35357926 10.1126/science.abf6805

[29] Kim, R. Y. et al. Oestrogen receptor β ligand acts on CD11c&plus; cells to mediate protection in experimental autoimmune encephalomyelitis. *Brain* **141**, 132–147 (2018). 29228214 10.1093/brain/awx315 PMC5837360

[30] Mayrhofer, F. et al. Reduction in CD11c(+) microglia correlates with clinical progression in chronic experimental autoimmune demyelination. *Neurobiol. Dis.* **161**, 105556 (2021). 34752925 10.1016/j.nbd.2021.105556

[31] Palma, A. et al. Clemastine induces an impairment in developmental myelination. *Front. Cell Dev. Biol.* **10**, 841548 (2022). 35372341 10.3389/fcell.2022.841548 PMC8970281

[32] Wlodarczyk, A. et al. CSF1R stimulation promotes increased neuroprotection by CD11c+ microglia in EAE. *Front. Cell Neurosci.* **12**, 523 (2018). 30687013 10.3389/fncel.2018.00523 PMC6335250

[33] Komine, O. et al. Innate immune adaptor TRIF deficiency accelerates disease progression of ALS mice with accumulation of aberrantly activated astrocytes. *Cell Death Differ.* **25**, 2130–2146 (2018). 29568058 10.1038/s41418-018-0098-3 PMC6261996

[34] Barichello, T., Simoes, L. R., Quevedo, J. & Zhang, X. Y. Microglial activation and psychotic disorders: evidence from pre-clinical and clinical studies. *Curr. Top Behav. Neurosci.* **44**, 161–205 (2020). 30828767 10.1007/7854_2018_81

[35] Piotrowska, A. et al. Pharmacological Blockade of Spinal CXCL3/CXCR2 Signaling by NVP CXCR2 20, a Selective CXCR2 antagonist, reduces neuropathic pain following peripheral nerve injury. *Front. Immunol.* **10**, 2198 (2019). 31616413 10.3389/fimmu.2019.02198 PMC6775284

[36] Rosin, J. M., Sinha, S., Biernaskie, J. & Kurrasch, D. M. A subpopulation of embryonic microglia respond to maternal stress and influence nearby neural progenitors. *Dev. Cell* **56**, 1326–1345.e1326 (2021). 10.1016/j.devcel.2021.03.018 33887203

[37] Serdar, M. et al. Involvement of CXCL1/CXCR2 during microglia activation following inflammation-sensitized hypoxic-ischemic brain injury in neonatal rats. *Front. Neurol.* **11**, 540878 (2020). 33123073 10.3389/fneur.2020.540878 PMC7573390

[38] Nomaki, K., Fujikawa, R., Masuda, T. & Tsuda, M. Spatiotemporal dynamics of the CD11c(+) microglial population in the mouse brain and spinal cord from developmental to adult stages. *Mol. Brain* **17**, 24 (2024). 38762724 10.1186/s13041-024-01098-2 PMC11102220

[39] Young, A. M., Campbell, E., Lynch, S., Suckling, J. & Powis, S. J. Aberrant NF-kappaB expression in autism spectrum condition: a mechanism for neuroinflammation. *Front. Psychiatry* **2**, 27 (2011). 21629840 10.3389/fpsyt.2011.00027 PMC3098713

[40] Zhu, Y. et al. Distinct phenotypes of inflammation associated macrophages and microglia in the prefrontal cortex schizophrenia compared to controls. *Front. Neurosci.* **16**, 858989 (2022). 35844224 10.3389/fnins.2022.858989 PMC9279891

[41] Paolicelli, R. C. et al. Microglia states and nomenclature: a field at its crossroads. *Neuron* **110**, 3458–3483 (2022). 36327895 10.1016/j.neuron.2022.10.020 PMC9999291

[42] Chen, Y. & Colonna, M. Microglia in Alzheimer’s disease at single-cell level. Are there common patterns in humans and mice? *J. Exp. Med*. **218**, e20202717 (2021). 10.1084/jem.20202717 PMC8302448 34292312

[43] Sobue, A. et al. Microglial gene signature reveals loss of homeostatic microglia associated with neurodegeneration of Alzheimer’s disease. *Acta Neuropathol. Commun.* **9**, 1 (2021). 33402227 10.1186/s40478-020-01099-x PMC7786928

[44] Jia, J. et al. CD11c(+) microglia promote white matter repair after ischemic stroke. *Cell Death Dis.* **14**, 156 (2023). 36828819 10.1038/s41419-023-05689-0 PMC9958101

[45] Hammond, T. R., Marsh, S. E. & Stevens, B. Immune signaling in neurodegeneration. *Immunity* **50**, 955–974 (2019). 30995509 10.1016/j.immuni.2019.03.016 PMC6822103

[46] Hammond, T. R. et al. Single-cell RNA sequencing of microglia throughout the mouse lifespan and in the injured brain reveals complex cell-state changes. *Immunity* **50**, 253–271.e256 (2019). 10.1016/j.immuni.2018.11.004 PMC6655561 30471926

[47] Stratoulias, V., Venero, J. L., Tremblay, M, E. & Joseph, B. Microglial subtypes: diversity within the microglial community. *EMBO J.* **38**, e101997 (2019). 31373067 10.15252/embj.2019101997 PMC6717890

[48] Peterson, B. S. et al. Using tissue microstructure and multimodal MRI to parse the phenotypic heterogeneity and cellular basis of autism spectrum disorder. *J. Child Psychol. Psychiatry* **63**, 855–870 (2022). 34762311 10.1111/jcpp.13531 PMC9091058

[49] Kútna, V., O’Leary, V. B., Hoschl, C. & Ovsepian, S. V. Cerebellar demyelination and neurodegeneration associated with mTORC1 hyperactivity may contribute to the developmental onset of autism-like neurobehavioral phenotype in a rat model. *Autism Res.* **15**, 791–805 (2022). 35178882 10.1002/aur.2688

[50] Linke, A. C. et al. Morphometry and functional connectivity of auditory cortex in school-age children with profound language disabilities: five comparative case studies. *Brain Cognit.* **155**, 105822 (2021). 34837801 10.1016/j.bandc.2021.105822

[51] Phan, B. N. et al. A myelin-related transcriptomic profile is shared by Pitt-Hopkins syndrome models and human autism spectrum disorder. *Nat. Neurosci.* **23**, 375–385 (2020). 32015540 10.1038/s41593-019-0578-x PMC7065955

[52] Sui, Y. V. et al. Diffusional kurtosis imaging of the corpus callosum in autism. *Mol. Autism* **9**, 62 (2018). 30559954 10.1186/s13229-018-0245-1 PMC6293510

[53] Temur, H. O. et al. Correlation between DTI findings and volume of corpus callosum in children with AUTISM. *Curr. Med. Imaging Rev.* **15**, 895–899 (2019). 32008536 10.2174/1573405614666181005114315

[54] Uccelli, N. A. et al. Neurobiological substrates underlying corpus callosum hypoconnectivity and brain metabolic patterns in the valproic acid rat model of autism spectrum disorder. *J. Neurochem.* **159**, 128–144 (2021). 34081798 10.1111/jnc.15444

[55] Zhang, X. F. et al. Poly(I:C) challenge alters brain expression of oligodendroglia-related genes of adult progeny in a mouse model of maternal immune activation. *Front. Mol. Neurosci.* **13**, 115 (2020). 32714147 10.3389/fnmol.2020.00115 PMC7340146

[56] Jain, V. G. et al. Acute histologic chorioamnionitis independently and directly increases the risk for brain abnormalities seen on magnetic resonance imaging in very preterm infants. *Am. J. Obstet. Gynecol.* **227**, 623.e621–623.e613 (2022). 10.1016/j.ajog.2022.05.042 PMC10008527 35644247

[57] Martini, S. et al. Neurodevelopmental correlates of brain magnetic resonance imaging abnormalities in extremely low-birth-weight infants. *J. Pediatr.* **262**, 113646 (2023). 37516269 10.1016/j.jpeds.2023.113646

[58] Rito, D. C., Viehl, L. T., Buchanan, P. M., Haridas, S. & Koenig, J. M. Augmented Th17-type immune responses in preterm neonates exposed to histologic chorioamnionitis. *Pediatr. Res.* **81**, 639–645 (2017). 27870827 10.1038/pr.2016.254 PMC5395318

[59] Iitani, Y. et al. Interleukin-17A stimulation induces alterations in Microglial microRNA expression profiles. *Pediatr. Res.* **95**, 167–173 (2024). 37758861 10.1038/s41390-023-02825-6

[60] Liu, Y. et al. Galectin-3 regulates microglial activation and promotes inflammation through TLR4/MyD88/NF-kB in experimental autoimmune uveitis. *Clin. Immunol.* **236**, 108939 (2022). 35121106 10.1016/j.clim.2022.108939

[61] Lively, S. & Schlichter, L. C. Microglia responses to pro-inflammatory stimuli (LPS, IFNγ+TNFα) and reprogramming by resolving cytokines (IL-4, IL-10). *Front. Cell Neurosci.* **12**, 215 (2018). 30087595 10.3389/fncel.2018.00215 PMC6066613

[62] Shen, X., Qiu, Y., Wight, A. E., Kim, H. J. & Cantor, H. Definition of a mouse microglial subset that regulates neuronal development and proinflammatory responses in the brain. *Proc. Natl Acad. Sci. USA* **119**, e2116241119 (2022). 10.1073/pnas.2116241119 PMC8872761 35177477

[63] Rayasam, A., Fukuzaki, Y. & Vexler, Z. S. Microglia-leucocyte axis in cerebral ischaemia and inflammation in the developing brain. *Acta Physiol.* **233**, e13674 (2021). 10.1111/apha.13674 PMC9093042 33991400

[64] Mülling, K. et al. Neutrophil dynamics, plasticity and function in acute neurodegeneration following neonatal hypoxia-ischemia. *Brain Behav. Immun.* **92**, 234–244 (2021). 33333168 10.1016/j.bbi.2020.12.012

[65] Walker, D. M. & Marlow, N. Neurocognitive outcome following fetal growth restriction. *Arch. Dis. Child Fetal Neonatal. Ed.* **93**, F322–F325 (2008). 18381841 10.1136/adc.2007.120485

[66] Fanous, M. et al. Quantifying myelin content in brain tissue using color Spatial Light Interference Microscopy (cSLIM). *PLoS ONE* **15**, e0241084 (2020). 33211727 10.1371/journal.pone.0241084 PMC7676665

[67] Komine, O. et al. Genetic background variation impacts microglial heterogeneity and disease progression in amyotrophic lateral sclerosis model mice. *iScience* **27**, 108872 (2024). 38318390 10.1016/j.isci.2024.108872 PMC10839647

[68] Kim, D., Paggi, J. M., Park, C., Bennett, C. & Salzberg, S. L. Graph-based genome alignment and genotyping with HISAT2 and HISAT-genotype. *Nat. Biotechnol.* **37**, 907–915 (2019). 31375807 10.1038/s41587-019-0201-4 PMC7605509

[69] Pertea, M. et al. StringTie enables improved reconstruction of a transcriptome from RNA-seq reads. *Nat. Biotechnol.* **33**, 290–295 (2015). 25690850 10.1038/nbt.3122 PMC4643835

[70] Robinson, M. D., McCarthy, D. J. & Smyth, G. K. edgeR: a Bioconductor package for differential expression analysis of digital gene expression data. *Bioinformatics* **26**, 139–140 (2010). 19910308 10.1093/bioinformatics/btp616 PMC2796818

[71] Storey, J. D. & Tibshirani, R. Statistical significance for genomewide studies. *Proc. Natl Acad. Sci. USA* **100**, 9440–9445 (2003). 12883005 10.1073/pnas.1530509100 PMC170937

[72] Ge, S. X., Son, E. W. & Yao, R. iDEP: an integrated web application for differential expression and pathway analysis of RNA-Seq data. *BMC Bioinform.* **19**, 534 (2018). 10.1186/s12859-018-2486-6 PMC6299935 30567491

[73] Semple, B. D., Blomgren, K., Gimlin, K., Ferriero, D. M. & Noble-Haeusslein, L. J. Brain development in rodents and humans: identifying benchmarks of maturation and vulnerability to injury across species. *Prog. Neurobiol.* **106-107**, 1–16 (2013). 23583307 10.1016/j.pneurobio.2013.04.001 PMC3737272

[74] Vincze, A., Mázló, M., Seress, L., Komoly, S. & Abrahám, H. A correlative light and electron microscopic study of postnatal myelination in the murine corpus callosum. *Int. J. Dev. Neurosci.* **26**, 575–584 (2008). 18556167 10.1016/j.ijdevneu.2008.05.003

[75] Pujol, J. et al. Delayed myelination in children with developmental delay detected by volumetric MRI. *Neuroimage* **22**, 897–903 (2004). 15193620 10.1016/j.neuroimage.2004.01.029

[76] Brouwer, M. J. et al. Sequential cranial ultrasound and cerebellar diffusion weighted imaging contribute to the early prognosis of neurodevelopmental outcome in preterm infants. *PLoS ONE* **9**, e109556 (2014). 25329772 10.1371/journal.pone.0109556 PMC4203729

[77] Omizzolo, C. et al. Neonatal brain abnormalities and memory and learning outcomes at 7 years in children born very preterm. *Memory* **22**, 605–615 (2014). 23805915 10.1080/09658211.2013.809765 PMC3965650

[78] Katsuki, S. et al. Hypertensive disorders of pregnancy and alterations in brain metabolites in preterm infants: a multi-voxel proton MR spectroscopy study. *Early Hum. Dev.* **163**, 105479 (2021). 34624700 10.1016/j.earlhumdev.2021.105479

[79] Ushida, T. et al. Impact of maternal hypertensive disorders of pregnancy on brain volumes at term-equivalent age in preterm infants: a voxel-based morphometry study. *Pregnancy Hypertens* **25**, 143–149 (2021). 34139669 10.1016/j.preghy.2021.06.003

[80] Blanc, W. A. Pathology of the placenta, membranes, and umbilical cord in bacterial, fungal, and viral infections in man. *Monogr. Pathol*. **22**, 67–132 (1981). 7024790

[81] Kidokoro, H., Neil, J. J. & Inder, T. E. New MR imaging assessment tool to define brain abnormalities in very preterm infants at term. *AJNR Am. J. Neuroradiol.* **34**, 2208–2214 (2013). 23620070 10.3174/ajnr.A3521 PMC4163698

[82] Fuma, K. et al. Antenatal corticosteroids-to-delivery interval associates cord blood S100B levels. *J. Obstet. Gynaecol. Res.* **49**, 1129–1136 (2023). 10.1111/jog.15582 36759328

[83] Velmeshev, D. et al. Single-cell genomics identifies cell type-specific molecular changes in autism. *Science* **364**, 685–689 (2019). 31097668 10.1126/science.aav8130 PMC7678724

[84] Zheng, G. X. et al. Massively parallel digital transcriptional profiling of single cells. *Nat. Commun.* **8**, 14049 (2017). 28091601 10.1038/ncomms14049 PMC5241818

[85] Hao, Y. et al. Integrated analysis of multimodal single-cell data. *Cell* **184**, 3573–3587.e3529 (2021). 10.1016/j.cell.2021.04.048 PMC8238499 34062119

[86] Korsunsky, I. et al. Fast, sensitive and accurate integration of single-cell data with Harmony. *Nat. Methods* **16**, 1289–1296 (2019). 31740819 10.1038/s41592-019-0619-0 PMC6884693

[87] Kanda, Y. Investigation of the freely available easy-to-use software ‘EZR’ for medical statistics. *Bone Marrow Transplant* **48**, 452–458 (2013). 23208313 10.1038/bmt.2012.244 PMC3590441

