# Supplementary material for: Prenatal inflammation impairs early CD11c-positive microglia induction and delays myelination in neurodevelopmental disorders
Source: Commun Biol. 2025 Jan 17;8:75. doi: 10.1038/s42003-025-07511-3 (PMC11742679; doi:10.1038/s42003-025-07511-3)
Supplement: Supplementary file 2 — Supplementary information [file 42003_2025_7511_MOESM2_ESM.pdf]

Supplementary information

Supplementary Fig. 1

Supplementary Fig. 2

Supplementary Fig. 3

Supplementary Methods

Supplementary Table 1

Supplementary Table 2

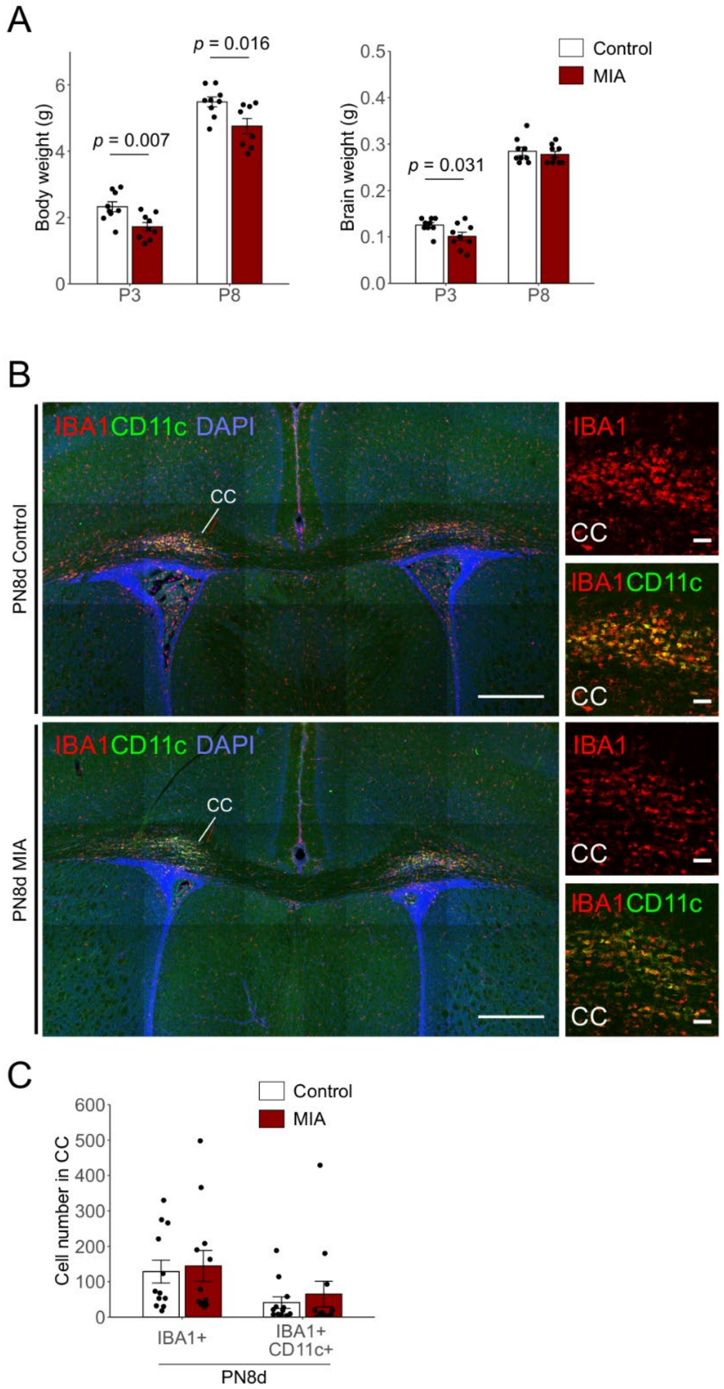

Supplementary Fig. 1

A. Body weights and brain weights of the pups at PN3d and PN8d (n = 8–9; maximum 2 offspring per litter).

B. Immunofluorescence staining of IBA1 and CD11c in the corpus callosum in PN8d in the control (upper panel) and MIA (lower panel) groups. Scale bar = 200µm (tiling image), 50µm (magnified image).

C. Cell numbers of $IBA1^+$ and $CD11c^+$ microglia in the corpus callosum in PN8d offspring (n = 6 from three litters in each group).

Data are presented as mean ± SEM. MIA, maternal immune activation. CC, corpus callosum.

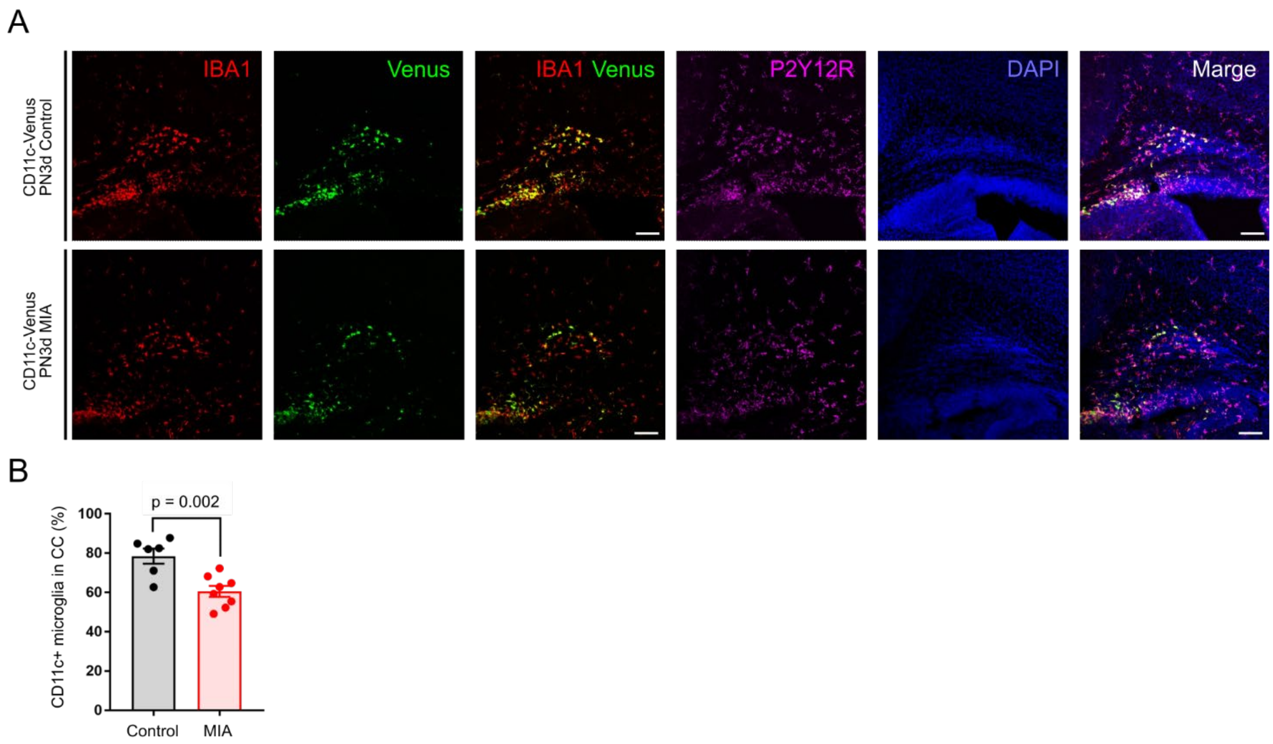

Supplementary Fig. 2

A. Immunofluorescence staining in the corpus callosum in PN3d *CD11c-Venus* mice in the control (upper panel) and MIA (lower panel) groups. Scale bar = 100µm.

B. Cell percentage of $IBA1^{+}Venus^{+}$ cells ($CD11c^{+}$ microglia). N = 6–8 offspring per group, one offspring per litter.

Data are presented as mean ± SEM. MIA, maternal immune activation. CC, corpus callosum.

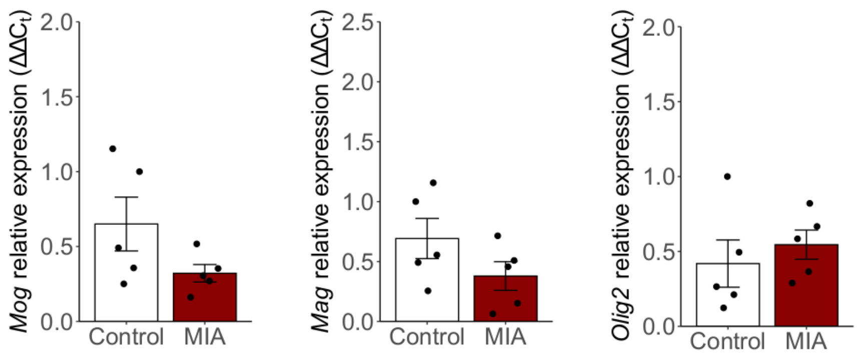

Supplementary Fig. 3

Mog, *Mag*, and *Olig2* mRNA expressions in PN8d offspring between the control and MIA groups (n = 5 in each group; maximum 2 offspring per litter).

Data are presented as mean ± SEM. MIA, maternal immune activation.

Supplementary Methods

(Methods for *CD11c-Venus* transgenic mice)

The animal protocol was approved by the Institutional Animal Care and Use committee review panels at Kyushu University and followed the relevant national and international guidelines contained in the 'Act on Welfare and Management of Animals' (Ministry of Environment of Japan) and 'Regulation of Laboratory Animals' (Kyushu University). All *CD11c-Venus* [B6.Cg-Tg(Itgax-Venus)1Mnz/J] mice were maintained under a standard specific pathogen-free environment with a 12 hours light/dark cycle and provided with free access to food and water. The MIA model was created in the same manner as for ICR mice. However, administration of 0.77 mg/kg of LPS to *CD11c-Venus* mice resulted in miscarriage in all cases. Therefore, the LPS dosage was reduced to 0.5 mg/kg.

PN3d offspring (n = 6–8 per group, one offspring per litter) were deeply anesthetized by hypothermia for 2–4 min until movement ceased, and perfused transcardially with PBS, followed by 4% PFA. Dissected brains were fixed in 4% PFA 4–6 hours at 4 °C and then cryoprotected with 30% sucrose at 4 °C. Tissues were embedded in OCT compound (Sakura Finetek, Japan), sectioned at a thickness of 50 µm, blocked with 5% BSA and 1% donkey serum in PBS, and permeabilized with 0.5% Triton X-100. The primary antibodies were added for 48 hours at 4 °C followed by secondary antibodies and DAPI staining for 2 hours at room temperature. The following primary antibodies were used: guinea pig anti-Iba1 (1:2000, 234 004; Synaptic systems), rabbit anti-P2Y12R (1:2000, AS-55043A, AnaSpec); The following secondary antibodies were used: donkey anti-guinea pig Alexa546 (1:2000, 706-165-148; Jackson immunoReseach), donkey anti-rabbit Alexa647 (1:2000, ab150063, abcam). Tissue sections were analyzed using LSM700 Imaging System (ZEN 2012, Carl Zeiss).

Eight coronal sections at corpus callosum (Bregma +0.5mm in Allen Brain Atlas) were examined from one PN3d brain. The mean percentage of $CD11c^+$ microglia was calculated using the formula: 100 × $IBA1^+Venus^+$ cells / $IBA1^+$ cells.

Supplementary Table 1. Maternal and neonatal characteristics on the cord blood analysis.

	non-HCA (n = 56)	HCA (n = 13)	*p*-value
**Maternal characteristics**			
Maternal age (years)	34.5 [16–43]	37 [22–43]	0.59
Primiparous	32 (57.1%)	4 (30.8%)	0.125
Gestational age at delivery (weeks)	30.7 [24.6–33.9]	32.0 [24.4–33.7]	0.96
Cesarean section	49 (87.5%)	11 (84.6%)	0.67
Antenatal corticosteroid treatment	35 (62.5%)	9 (69.2%)	0.76
Antenatal magnesium sulfate treatment	17 (30.4%)	5 (38.5%)	0.74
**Neonatal characteristics**			
Male	32 (57.1%)	7 (53.8%)	0.83†
Birth weight (g)	1358.0 ± 468.3	1509.5 ± 561.6	0.32
Small for gestational age	14 (25.0%)	0 (0.0%)	0.06
Respiratory distress syndrome	30 (53.6%)	7 (53.8%)	0.99†
Duration of intubation (days)	1 [0–89]	1 [0–67]	0.48
Bronchopulmonary dysplasia	13 (23.2%)	2 (15.4%)	0.72
Intraventricular hemorrhage (grade 1 or 2)	2 (3.6%)	0 (0.0%)	>0.99
Patent ductus arteriosus ligation	1 (1.8%)	1 (7.7%)	0.34
Inotrope use	3 (5.4%)	0 (0.0%)	>0.99
Postnatal steroid use	6 (10.7%)	1 (7.7%)	>0.99
Necrotizing enterocolitis	0 (0.0%)	0 (0.0%)	ND
Infection	1 (1.8%)	2 (15.4%)	0.09
Treated retinopathy of prematurity	3 (5.4%)	1 (7.7%)	0.58
Duration of hospitalization (days)	60.5 [24–159]	47 [28–135]	0.37

Continuous variables are presented as mean ± SD or median [minimum–maximum] and *p*-values were calculated by Student's *t*-test or Mann–Whitney *U* test for normal or non-normal distribution, respectively. Categorical variables are presented as numbers (%) and *p*-values were calculated by Fisher's exact test or †χ^2 test as appropriate. HCA, histological chorioamnionitis; ND, not detected.

Supplementary Table 2. The list of primers for qRT-PCR.

Genes	Primer sequences	
	Forward	Reverse
Actb	CGTGGGCCGCCCTAGGCACCA	ACACGCAGCTCATTGTA
Ccl3	CTGACAAGCTCACCCTCTGT	TTCTCTTAGTCAGGAAAATGACACC
Cxcl1	ACCCAAACCGAAGTCATAGCC	TTGTCAGAAGCCAGCGTTCA
Cxcl10	CCACGTGTTGAGATCATTGCC	GAGGCTCTCTGCTGTCCATC
Il1b	CATCCAGCTTCAAATCTCGCAG	CACACACCAGCAGGTTATCATC
Lgals3	CAACAGGAGAGTCATTGTGTGTAA	TTCAACCAGGACTTGTATTTTGAAT
Cd11c	CTGGATAGCCTTTCTTCTGCTG	GCACACTGTGTCCGAACTCA
Plp	GCTTTCCCTGGCAAGGTTTG	TGAAGGTGAGCAGGGAAACT
Mbp	CACACGAGAACTACCCATTATGGC	GTGTTCGAGGTGTCACAATGTTCT
Mog	TCATGCAGCTATGCAGGACAA	TTTCGGTAGAGGTGAACCACT
Mag	CTGCCGCTGTTTTGGATAATGA	CATCGGGGAAGTCGAAACGG
Olig2	TCCACCAAGAAAGACAAGAAGCAGA	ATGGCGATGTTGAGGTCGTGC
