# Supplementary material for: Prenatal inflammation impairs early CD11c-positive microglia induction and delays myelination in neurodevelopmental disorders
Source: Commun Biol. 2025 Jan 17;8:75. doi: 10.1038/s42003-025-07511-3 (PMC11742679; doi:10.1038/s42003-025-07511-3)
Supplement: Supplementary file 5 — Reporting Summary [file 42003_2025_7511_MOESM5_ESM.pdf]

nature portfolio

Corresponding author(s): Tomomi Kotani

Last updated by author(s): Jan 2, 2025

Reporting Summary

Nature Portfolio wishes to improve the reproducibility of the work that we publish. This form provides structure for consistency and transparency in reporting. For further information on Nature Portfolio policies, see our Editorial Policies and the Editorial Policy Checklist.

Statistics

For all statistical analyses, confirm that the following items are present in the figure legend, table legend, main text, or Methods section.

n/a	Confirmed	
☐	☒	The exact sample size (*n*) for each experimental group/condition, given as a discrete number and unit of measurement
☐	☒	A statement on whether measurements were taken from distinct samples or whether the same sample was measured repeatedly
☐	☒	The statistical test(s) used AND whether they are one- or two-sided<br>*Only common tests should be described solely by name; describe more complex techniques in the Methods section.*
☐	☒	A description of all covariates tested
☐	☒	A description of any assumptions or corrections, such as tests of normality and adjustment for multiple comparisons
☐	☒	A full description of the statistical parameters including central tendency (e.g. means) or other basic estimates (e.g. regression coefficient) AND variation (e.g. standard deviation) or associated estimates of uncertainty (e.g. confidence intervals)
☐	☒	For null hypothesis testing, the test statistic (e.g. *F*, *t*, *r*) with confidence intervals, effect sizes, degrees of freedom and *P* value noted<br>*Give P values as exact values whenever suitable.*
☒	☐	For Bayesian analysis, information on the choice of priors and Markov chain Monte Carlo settings
☒	☐	For hierarchical and complex designs, identification of the appropriate level for tests and full reporting of outcomes
☒	☐	Estimates of effect sizes (e.g. Cohen's *d*, Pearson's *r*), indicating how they were calculated

Our web collection on statistics for biologists contains articles on many of the points above.

Software and code

Policy information about availability of computer code

Data collection	No custom algorithms or software was used.
Data analysis	No custom algorithms or software was used.

For manuscripts utilizing custom algorithms or software that are central to the research but not yet described in published literature, software must be made available to editors and reviewers. We strongly encourage code deposition in a community repository (e.g. GitHub). See the Nature Portfolio guidelines for submitting code & software for further information.

Data

Policy information about availability of data

All manuscripts must include a data availability statement. This statement should provide the following information, where applicable:

- Accession codes, unique identifiers, or web links for publicly available datasets
- A description of any restrictions on data availability
- For clinical datasets or third party data, please ensure that the statement adheres to our policy

The RNA-seq data have been deposited with links to BioProject accession number PRJDB15535 in the DDBJ BioProject database. All data supporting the findings of this study are available within the paper and its Supplementary Data. Other data supporting the findings of this study are available from the corresponding author (TK) upon reasonable request.

March 2021

Human research participants

Policy information about studies involving human research participants and Sex and Gender in Research.

Reporting on sex and gender	The sex of the neonates was determined biologically and reported.
Population characteristics	Gestational age and birth weight z-scores were used as covariates.
Recruitment	Cord blood collection was not performed for patients without consent, which may have resulted in a sample biased toward patients favorable to participation in the study. Additionally, participants for whom MRI acquisition at clinically appropriate time points or cord blood collection was difficult were excluded, leading to a sample biased toward relatively mild preterm infants.
Ethics oversight	the Institutional Ethics Committee of Nagoya University Hospital (approval numbers: 2015-0068 and 2018–0026)

Note that full information on the approval of the study protocol must also be provided in the manuscript.

Field-specific reporting

Please select the one below that is the best fit for your research. If you are not sure, read the appropriate sections before making your selection.

☒ Life sciences ☐ Behavioural & social sciences ☐ Ecological, evolutionary & environmental sciences

For a reference copy of the document with all sections, see nature.com/documents/nr-reporting-summary-flat.pdf

Life sciences study design

All studies must disclose on these points even when the disclosure is negative.

Sample size	A typical sample size for animal experiments (n = 3–8) was used. In Human study, all patients who were retrospectively available for analysis at the start of the study were included. Additionally, the sample size was increased in the revised manuscript in response to reviewer requests.
Data exclusions	All data obtained from the animal experiments were analyzed. In Human study, the exclusion criteria were largely pre-established. High-urgency cases (e.g., uterine rupture), which could introduce bias, were excluded individually based on consensus among multiple researchers.
Replication	One of the main findings of this study, the reduction of CD11c microglia in the corpus callosum of mice in the MIA group as observed through imaging, was replicated in a different laboratory.
Randomization	The allocation of animals was performed randomly.
Blinding	Due to the difficulty of the experiments, blinding of group allocation for animals was not performed. In the human study, the assessment of delayed myelination in newborns was conducted in a blinded manner.

Reporting for specific materials, systems and methods

We require information from authors about some types of materials, experimental systems and methods used in many studies. Here, indicate whether each material, system or method listed is relevant to your study. If you are not sure if a list item applies to your research, read the appropriate section before selecting a response.

Materials & experimental systems

n/a	Involved in the study	
☐	☒	Antibodies
☒	☐	Eukaryotic cell lines
☒	☐	Palaeontology and archaeology
☐	☒	Animals and other organisms
☒	☐	Clinical data
☒	☐	Dual use research of concern

Methods

n/a	Involved in the study	
☒	☐	ChIP-seq
☒	☐	Flow cytometry
☒	☐	MRI-based neuroimaging

Antibodies

Antibodies used	Rabbit monoclonal anti-PLP (myelin proteolipid protein, 1:200, #ab105784; Abcam plc, Cambridge, UK); rabbit polyclonal anti-MBP (myelin basic protein, 1:200, #AB980; Millipore, Temecula, CA, USA); rabbit anti-Iba1 (1:500, #019-19741; Wako); Armenian hamster anti-CD11c (1:10, #550283; BD Biosciences); rat anti-CLEC7A (1:50, #mabg-mdect-2; InvivoGen, San Diego, CA, USA); guinea pig anti-Iba1 (1:2000, 234 004; Synaptic systems); rabbit anti-P2Y12R (1:2000, AS-55043A, AnaSpec); goat anti-rabbit Alexa568 (1:500, #A11036; Invitrogen); goat anti-Hamster DyLight488 (1:500, #405503, BioLegend); goat anti-rat Alexa647 (1:500, #A21247; Invitrogen); donkey anti-guinea pig Alexa546 (1:2000, 706-165-148; Jackson immunoReseach); and donkey anti-rabbit Alexa647 (1:2000, ab150063, abcam).
Validation	The specificity of the antibodies was guaranteed by the manufacturer.

Animals and other research organisms

Policy information about studies involving animals; ARRIVE guidelines recommended for reporting animal research, and Sex and Gender in Research

Laboratory animals	Slc:ICR mice, pregnant, 8-9 weeks.
Wild animals	We did not involve wild animals.
Reporting on sex	Due to the difficulty of the experiments, the sex of the offspring was not considered.
Field-collected samples	We did not involve sample collected from the field.
Ethics oversight	The animal protocols used in this study were approved by the Animal Experiment Committee of Nagoya University (approval number: M220211-001) and adhered to the relevant guidelines and regulations.

Note that full information on the approval of the study protocol must also be provided in the manuscript.
