# Supplementary material for: Prenatal inflammation impairs early CD11c-positive microglia induction and delays myelination in neurodevelopmental disorders
Source: Commun Biol. 2025 Jan 17;8:75. doi: 10.1038/s42003-025-07511-3 (PMC11742679; doi:10.1038/s42003-025-07511-3)
Supplement: Supplementary file 1 — Transparent Peer Review file [file 42003_2025_7511_MOESM1_ESM.pdf]

nature portfolio

Peer Review File

Prenatal Inflammation Impairs Early CD11c-Positive Microglia Induction and Delays Myelination in Neurodevelopmental Disorders

Corresponding Author: Dr Tomomi Kotani

This manuscript has been previously reviewed at another journal. This document only contains information relating to versions considered at Communications Biology.

Open Access This Peer Review File is licensed under a Creative Commons Attribution 4.0 International License, which permits use, sharing, adaptation, distribution and reproduction in any medium or format, as long as you give appropriate credit to the original author(s) and the source, provide a link to the Creative Commons license, and indicate if changes were made.
In cases where reviewers are anonymous, credit should be given to 'Anonymous Referee' and the source.
The images or other third party material in this Peer Review File are included in the article's Creative Commons license, unless indicated otherwise in a credit line to the material. If material is not included in the article's Creative Commons license and your intended use is not permitted by statutory regulation or exceeds the permitted use, you will need to obtain permission directly from the copyright holder.
To view a copy of this license, visit https://creativecommons.org/licenses/by/4.0/
